# Biodegradable Ruthenium‐Rhenium Complexes Containing Nanoamplifiers: Triggering ROS‐Induced CO Release for Synergistic Cancer Treatment

**DOI:** 10.1002/advs.202403795

**Published:** 2024-07-12

**Authors:** Aijie Liu, Zhenkun Huang, Xiangfu Du, Naresh Duvva, Yuting Du, Zihao Teng, Zhihuan Liao, Chen Liu, Haining Tian, Shuaidong Huo

**Affiliations:** ^1^ State Key Laboratory of Cellular Stress Biology Fujian Provincial Key Laboratory of Innovative Drug Target Research School of Pharmaceutical Sciences Xiamen University Xiamen Fujian 361102 China; ^2^ Shenzhen Research Institute of Xiamen University Shenzhen Guangdong 518057 China; ^3^ Department of Chemistry‐Ångström Laboratory Box 523 Uppsala University Uppsala SE‐75120 Sweden

**Keywords:** cascaded nanoamplifier, gas therapy, photodynamic therapy, ROS‐induced CO release, ruthenium‐rhenium complexes copolymer

## Abstract

The constrained effectiveness of photodynamic therapy (PDT) has impeded its widespread use in clinical practice. Urgent efforts are needed to address the shortcomings faced in photodynamic therapy, such as photosensitizer toxicity, short half‐life, and limited action range of reactive oxygen species (ROS). In this study, a biodegradable copolymer nanoamplifier is reported that contains ruthenium complex (Ru‐complex) as photosensitizer (PS) and rhenium complex (Re‐complex) as carbon monoxide (CO)‐release molecule (CORM). The well‐designed nanoamplifier brings PS and CORM into close spatial proximity, significantly promotes the utilization of light‐stimulated reactive oxygen species (ROS), and cascaded amplifying CO release, thus enabling an enhanced synergistic effect of PDT and gas therapy for cancer treatment. Moreover, owing to its intrinsic photodegradable nature, the nanoamplifier exhibits good tumor accumulation and penetration ability, and excellent biocompatibility in vivo. These findings suggest that the biodegradable cascaded nanoamplifiers pave the way for a synergistic and clinically viable integration of photodynamic and gas therapy.

## Graphical abstract

This study introduces a biodegradable copolymer nanoamplifier containing ruthenium (Ru) complexes and rhenium (Re) complexes to generate ROS and cascade‐amplified carbon monoxide (CO) release under illumination. The significance of the proximity of Ru and Re‐complexes is observed both extracellularly and intracellularly. Owing to its inherent photodegradable nature, the nanoamplifier exhibits outstanding biocompatibility, and effective accumulation and penetration into tumor, facilitating synergistic therapy.

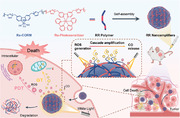

## 1 Introduction

Phototherapy, as a therapeutic modality, relies primarily on exposing patients to light to achieve its therapeutic effects.^[^ ^1^, ^2^ ^]^ Over the years, extensive research has matured phototherapy into a well‐established clinical treatment method.^[^ ^3^, ^4^, ^5^ ^]^ Among emerging phototherapy techniques, photodynamic therapy (PDT) stands out due to its high success rate, non‐invasiveness, minimal toxicity, and side effects, effectively addressing challenges conventional cancer treatment methods face.^[^ ^6^, ^7^, ^8^ ^]^

The function of PDT relies on photosensitizers (PSs) absorbing incident light, transitioning from the ground state to a short‐lived (≈ns) excited singlet state, and then undergoing an intersystem transition to a relatively more stable (≈ms) excited triplet state. This excited triplet state of PSs (^3^PS*) can return to the ground state through Type I or Type II photodynamic reactions. In Type I, the activated PSs transfer excited electrons to substrates, forming various reactive oxygen species (ROS), including superoxide(O₂^−^), hydroxyl radical (HO^•^). In Type II, triplet‐triplet energy transfer (TTEnT) from the ^3^PS* to triplet oxygen (^3^O₂) forms highly reactive singlet oxygen (¹O₂).^[^ ^9^, ^10^ ^]^ Large amounts of local ROS ultimately induce various cell death pathways and limit the nutrients’ arrival by irreversibly damaging tumor vasculature.^[^ ^11^ ^]^ However, the current therapeutic potential of PDT is significantly limited by the short lifespan and narrow action distance of ROS.^[^ ^12^ ^]^ For example, the lifetime of ¹O₂ in cells ranges from 0.01 to 45 µs, restricting the action radius of ≈10 to 270 nm.^[^ ^13^, ^14^, ^15^ ^]^ As a consequence, photodamage is only confined to regions where photosensitizers localize, limiting the therapeutic efficacy of PDT.^[^ ^16^ ^]^ Therefore, enhancing the action duration and distance of ROS is crucial for its potential clinical transformation.^[^ ^5^, ^12^, ^17^ ^]^

Carbon monoxide (CO), recognized as a crucial endogenous biological signaling molecule,^[^ ^18^, ^19^, ^20^ ^]^ has emerged as a promising gas therapeutic reagent for cancer treatment with negligible side effects.^[^ ^21^, ^22^ ^]^ Elevated local concentration of CO has demonstrated superior cell‐killing capabilities by accelerating mitochondrial respiration, triggering metabolic exhaustion, and inducing mitochondrial dysfunction.^[^ ^23^, ^24^, ^25^ ^]^ Throughout the last few decades, carbon monoxide‐releasing molecules (CORMs) have been developed through the coordinating of CO to transition‐metal centers, providing a controlled and safer alternative to the administration of gaseous CO.^[^ ^26^, ^27^ ^]^ Among these, rhenium (Re) metal‐centered CORM has exhibited exceptional stability and biocompatibility. The controlled release of CO is easily achieved through light activation.^[^ ^28^ ^]^ Therefore, harnessing the properties of CO, such as its long half‐life and extensive action distance, can be an effective strategy to overcome the limitations of PDT. This positions the potential synergistic effects of ROS and CO as a formidable combination, offering an efficient approach to cancer treatment.^[^ ^29^ ^]^

**Scheme 1:**
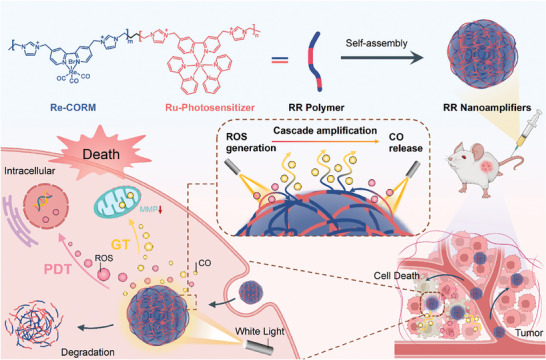
Illustration of biodegradable polymer‐based cascaded nanoamplifiers stimulating ROS‐induced CO release for synergistic treatment under light.

In pursuit of the proposed synergistic strategy that amalgamates PDT and gas therapy (GT) to effectively eradicate tumors, we herein developed biodegradable Ru‐Re‐complexes containing copolymer (referred to as RR polymer)‐based cascaded nanoamplifiers capable of generating photoinduced ROS and cascaded amplifying CO release for synergistic cancer treatment (**Scheme** **1**). A Ruthenium (Ru)‐complex was chosen as the PS and ROS generator, which is a classic photosensitizer that has garnered significant attention due to its distinctive optical properties. It has a relatively long excited state lifetime of approximately a few hundred nanoseconds, enabling it to generate a high amount of singlet oxygen (^1^O_2_) through a TTEnT process from the excited ^3^PS* to the triplet oxygen (^3^O_2_). A Re‐complex was selected as a CORM due to its exceptional stability in the dark and gradual release of CO under light stimulation.^[^ ^30^ ^]^ For the first time, we demonstrated that the CO‐releasing rate can be significantly accelerated in the presence of ROS compared to light alone. This acceleration is contributed to the spatial proximity design of copolymer, which facilitates the delivery path of ROS from the Ru‐complex to the nearby Re‐complex, enhancing the ROS utilization and boosting the controlled CO release. Imidazole was applied to simplify the co‐polymerization reaction. Additionally, the positively charged nature of imidazole enabled higher water dispersibility and cellular uptake efficiency of RR polymer‐formed nanoparticles (referred to as RR NPs). Under the white light irradiation, the excited Ru‐complex generates an abundance of ROS, which in turn, destabilizes the binding between the CO ligand and Re metal center, promptly inducing CO release and initiating a cascaded amplification effect. The concerted action of ROS and CO results in damage to cellular nuclei and mitochondria, significantly enhancing the therapeutic efficacy. This new approach concurrently achieves light‐regulated PDT and GT, intending to impart a transformative impact on cancer treatment.

## 2 Results and Discussion

### 2.1 Preparation and Characterization of Nanoamplifiers

**Figure 1:**
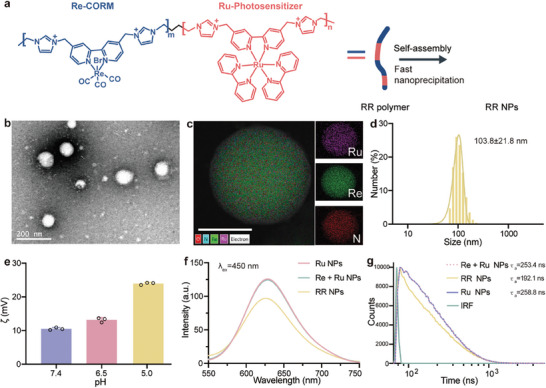
Preparation and characterization of RR NPs. a) Molecular formula of RR polymer and synthesis route of RR NPs. b) TEM image of RR NPs. c) EDS elemental mapping of RR NPs, (Scale bar, 50 nm). d) The hydrodynamic size of RR NPs (n = 3 independent experiments). e) The Zeta potential (*ζ*) of RR NPs at various pH (n = 3 independent experiments). f) Fluorescence spectrum of Ru NPs, Ru + Re blended NPs and RR NPs. g) Time‐resolved single‐photon counting (TCSPC) analysis of Ru NPs, Re + Ru blended NPs and RR NPs, λ_ex_=485 nm, λ_em_=625 nm.

The preparation of nanoamplifier RR NPs was carried out in two steps. First, RR polymers were synthesized via atomic transfer radical polymerization (ATRP), with detailed synthetic procedures presented in the supporting information (SI). Parallelly, Re‐polymer and Ru‐polymer were also synthesized using a similar methodology. The chemical structures of all polymers were confirmed via ^1^H NMR analysis (Figures S1–S11, Supporting Information). Inductively coupled plasma mass spectrometry (ICP‐MS) results confirmed a close to 1:1 ratio of Re and Ru elements in RR polymer (Table S1, Supporting Information), aligning with the feeding ratio. **Figure** **1**a illustrates the chemical structure of RR polymer and the preparation of RR NPs using flash‐precipitation. Additionally, for comparative analysis, Re NPs, Ru NPs, and Re + Ru blended NPs derived from Re‐polymer, Ru‐polymer, and a mixture of Re‐polymer/Ru‐polymer, respectively, were also prepared. All NPs displayed a uniform size distribution, ≈120 nm in diameter, as confirmed by the dynamic light scattering (DLS) and transmission electron microscope (TEM) analysis, shown in Figures S12,S13 (Supporting Information), respectively. TEM observation demonstrated that RR NPs have a typical spherical morphology (Figure 1b). Energy‐dispersive X‐ray spectroscopy (EDS) analysis confirmed the elemental composition, revealing a homogeneous and closely localized arrangement of Re and Ru elements in RR NPs (Figure 1c). DLS results show an average diameter of 103.8 ± 21.8 nm (Figure 1d), consistent with the above observations. RR NPs are stable for long‐term storage, no significant changes were found in size as well as the polydisperse index (PDI) in various media (Figure S14, Supporting Information), including water, phosphate‐buffered saline (PBS), and Dulbecco's Modified Eagle Medium (DMEM). In addition, their morphology and size remained the same after 1 month storage in a dark environment (Figure S15, Supporting Information). Blended NPs containing a mixture of Re‐polymer and Ru‐polymer exhibited a core–shell‐like structure (Figure S16, Supporting Information), suggesting a phase separation between these two positively charged polymers. The significance of this proximate localization of these two complexes in RR NPs was corroborated in the subsequent CO assay. The cationic linkers of imidazole groups endow RR NPs with higher positive surface potential under lower pH conditions (Figure 1e), offering advantages for subsequent tumor accumulation and cellular uptake.

Subsequently, a comprehensive characterization of the optical properties of RR NPs was conducted. The absorption spectrum of RR NPs was similar to Ru‐polymer/Re‐polymer blended NPs (Re + Ru NPs), as shown in Figure S17 (Supporting Information). Under the excitation of 450 nm, RR NPs, Ru NPs, and Re + Ru NPs exhibited a photoluminescent peak at ≈625 nm (Figure 1f), characteristic of the triplet metal‐to‐ligand charge‐transfer ^3^(MLCT) excited‐state of Ru‐complex.^[^ ^31^ ^]^ In contrast, only negligible photoluminescence (PL) intensity was observed for Re NPs under this excitation wavelength (Figure S18, Supporting Information). Maintaining an equivalent molar concentration of Ru‐complex, the photoluminescent intensity of RR NPs was discovered to be lower than that of Ru NPs and Re + Ru NPs, accompanied by a quenched lifetime, as revealed by time‐resolved single‐photon counting (TCSPC) results (Figure 1g). This decline is attributed to the covalent linking of Re‐complex and Ru‐complex within RR polymer, facilitating electron transfer (ET) from the excited state of the Ru‐complex to the Re‐complex. This phenomenon is supported by the redox potential of the Re/Ru‐complex^[^ ^32^, ^33^ ^]^ and their respective polymers (Figure S19, Supporting Information). However, the quenching efficiency of Ru‐complex by Re‐complex is low, ≈24%, meaning that the majority of excited Ru‐complex in RR polymer still remains.

### 2.2 Assessment of ROS Generation and Controlled CO Release of Nanoamplifiers

**Figure 2:**
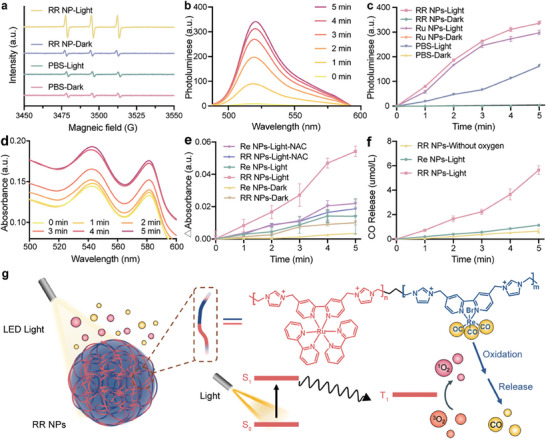
Assessment of ROS generation and controlled CO release of RR NPs. a) ESR spectrum of RR NPs co‐incubated with TEMP with and without light. b) Fluorescence spectrum of RR NPs co‐incubated with DCFH, with and without light treatment. c) The time‐dependent curves of the fluorescence intensity at the emission wavelength of 525 nm in different experimental groups (n = 3 independent experiments). d) UV–vis absorption spectra of RR NPs co‐incubated with myoglobin. e) The differential absorption intensity at 540 nm was recorded (n = 3 independent experiments). f) Determination of CO release of RR NPs by gas chromatography (n = 3 independent experiments). g) Principle of cascade amplification of RR NPs.

To explore the properties and functions of the nanoamplifier RR NPs, a comprehensive characterization of their light‐mediated ROS generation ability and CO release efficiency was conducted. First, the ROS generation of RR NPs was examined under white light irradiation and evaluated by using electron spin resonance (ESR) spectroscopy with 2,2,6,6‐Tetramethylpiperidine (TEMP) as a probe. Under the white light irradiation, a prominent ^1^O_2_ signal (1 : 1 : 1) was observed, and the signal intensity for RR NPs‐light group was approximately seven times higher than that of PBS‐Dark, PBS‐Light and RR NPs‐Dark groups. Slight signals observed in those control groups are due to the oxidation of TEMP by the dissolved oxygen in water aqueous,^[^ ^34^ ^]^ as shown in **Figure** **2**a. Furthermore, a quantitative assessment of ^1^O_2_ production efficiency was conducted by using a commercialized ^1^O_2_ quantification probe, 2,7‐dichlorodihydrofluorescein (DCFH). The fluorescent intensity of DCFH at 520 nm increased gradually in the presence of RR NPs due to its continuous reactions with the generated ^1^O_2_ (Figure 2b). When maintaining the concentration of the Ru‐complex at a constant level, the ^1^O_2‐_generating ability of RR NPs was found to be slightly higher (1.1 times) than Ru NPs, attributed to the inherent ROS‐generating capability of the Re‐complex (Figure S20, Supporting Information), but is considered negligible. No ROS was observed for all samples in the dark (Figure 2c).

Subsequently, a CO‐releasing assay was conducted to validate the proposed cascaded amplification capacity of RR NPs. A myoglobin (Mb) assay was utilized to confirm the CO release, the increasing absorption intensity at 540 nm is directly proportional to the concentration of CO‐bonded myoglobin, which can be used to calculate the released CO.^[^ ^35^ ^]^ As shown in Figure 2d, a notable enhancement of the characteristic absorption peak at 540 nm along with an extended irradiation time was observed, indicating an efficient CO release from RR NPs. Interestingly, the CO‐releasing rate of RR NPs was three times higher than that of Re NPs under white light irradiation (Figure 2e). This significant CO‐releasing rate of RR NPs could be due to the following possible mechanisms: 1) Efficient ET from the excited Ru‐complex to Re‐complex resulted in reduced Re‐complex, which makes it easier for CO ligand to dissociate and release;^[^ ^36^ ^]^ 2) ^1^O_2_ generated by the excited Ru‐complex could destabilize the nearby Re‐complex and accelerate the CO release. No significant CO release was observed for Re NPs and RR NPs without light irradiation (Figure 2e; Figure S21, Supporting Information).

To further elucidate the underlying mechanism governing the effective release of CO from RR NPs, N‐acetylcysteine (NAC), a typical ROS scavenger, can also be utilized as a sacrificial electron donor,^[^ ^37^, ^38^, ^39^ ^]^ was introduced into the NPs‐light groups. As shown in Figure 2e, the addition of NAC resulted in a slight increase of absorption at 540 nm for Re NPs‐light‐NAC group compared to Re NPs‐light group. In this scenario, NAC acts as a sacrificial electron donor, reducing the Re‐complex species and promoting their CO‐ligand dissociation.^[^ ^36^ ^]^ However, an opposing phenomenon was observed for RR NPs. The addition of NAC led to a significant decrease in the characteristic signal for the RR NPs‐light‐NAC group compared to the RR NPs‐light group. For RR NPs, both the ^1^O_2_ generated by the excited Ru‐complex and the ET process from the excited Ru‐complex can accelerate the CO release from the Re‐complex under irradiation. The introduction of NAC could effectively eliminate ROS and increase the quantity of the reduced Re‐complex species. The above results indicate that the ability of ^1^O_2_ to accelerate CO release outcompetes the ET process, which confirms that ^1^O_2_ is the primary factor contributing to the significantly enhanced CO release capacity of RR NPs.

Moreover, gas chromatography (GC) was applied to quantitatively evaluate the CO release from RR NPs. After 5 min of irradiation, the CO‐releasing efficiency of RR NPs was ≈17.3% (Figure 2f), more than twice that of the Re + Ru blended NPs that contain a mixture of Re‐polymer and Ru‐polymer (Figure S22, Supporting Information). This quantitive analysis provides additional evidence that the copolymerization of Re‐complex and Ru‐complex in RR NPs contributes to improving the utilization of the generated ^1^O_2_ from the Ru‐complex by the proximately localized Re‐complex, resulting in ROS‐boosted CO release mechanism.

The above results confirmed that the synthesized nanoamplifier RR NPs can generate ROS with enhanced CO release when exposed to light. The ET process from the excited Ru‐complex to the Re‐complex contributes to a portion of the CO release (Figure 2g). The amplification of CO release occurs through the following processes activated by the ^1^O_2_: 1) Upon exposure to white light, the PS was excited and formed a triplet state (^3^PS*), followed by TTEnT from ^3^PS* to ^3^O_2_ to form reactive ^1^O_2_; 2) The generated ^1^O_2_ was effectively utilized by the nearby Re‐complex, resulting in accelerated CO‐ligand dissociation and accomplishing the cascaded amplification. The synergistic interplay of ROS and CO would potentially advance cancer therapy.

### 2.3 Biocompatibility and Biodegradability Assessment of Nanoamplifiers

**Figure 3:**
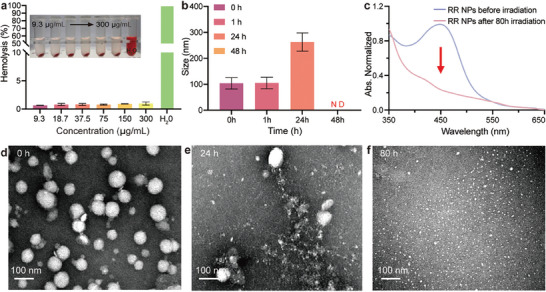
Biocompatibility and biodegradability of RR NPs. a) In vitro hemolysis percentage of RR NPs incubated at 37 °C for 6 h (n = 3 independent experiments). b) DLS analysis of size variation of RR NPs under the irradiation of 0, 1, 24, and 48 h (n = 3 independent experiments). ND means no determined. c) UV–vis absorption spectra of RR NPs pre‐ and post‐light irradiation. TEM images of RR NPs under the irradiation of d) 0 h, e) 24 h, and f) 80 h.

Before using the cascaded nanoamplifiers for tumor treatment, we validated their biocompatibility and biodegradability. Red blood cell hemolysis assay was first chosen for the preliminary biocompatibility evaluation of the RR NPs. As shown in **Figure** **3**a, the hemolysis rates of nanoamplifier RR NPs are <1.5% even at a high dose of 300 µg mL^−1^, in accordance with the American Society for Testing and Material Standard (ASTM F756‐08).^[^ ^40^ ^]^ This result underscores that the introduction of RR NPs does not induce hemolysis or provoke adverse reactions upon intravenous administration, affirming its commendable biocompatibility.

Considering the main components, Ru‐complex and Re‐complex of RR NPs can generate ^1^O_2_ and release CO under light exposure, the as‐prepared RR NPs exhibit an intrinsic biodegradability under irradiation, which can ensure their efficient clearance from the body after fulfilling their therapeutic administration. To confirm the degradability of RR NPs, the morphologies and optical properties of RR NPs under various light exposure durations were monitored. The DLS study revealed that the size of RR NPs initially increased within the first 24 h of irradiation, likely due to the swelling or aggregation of partially degraded particles. Importantly, prolonged irradiation (48 h) resulted in complete degradation of the nanoparticles (Figure 3b), consistent with UV–vis analysis showing the disappearance of the characteristic peak of RR NPs (Figure 3c). The time‐dependent TEM observation also indicated significant morphological changes after light irradiation (Figure 3d–f), further confirming the degradation of the RR NPs. This cumulative evidence not only establishes the robust biocompatibility and advantageous biodegradability of the RR NPs but also endows them with potential excellent light‐responsive tumor accumulation and penetration performance.^[^ ^41^, ^42^ ^]^

### 2.4 Cellular Uptake, ROS Generation, and CO Release Assessment of Nanoamplifiers

**Figure 4:**
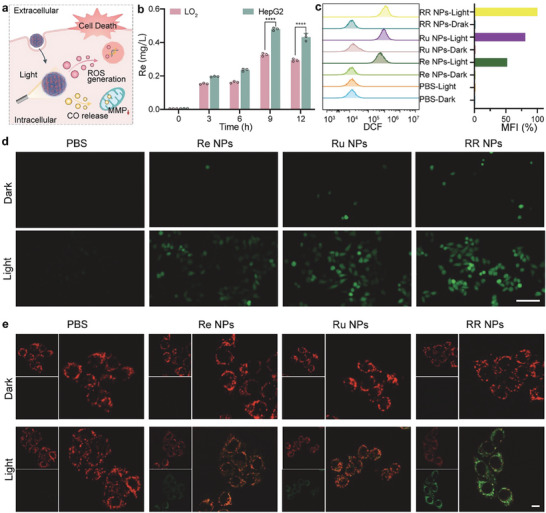
Cellular uptake, ROS generation, and CO release assessment of RR NPs. a) Schematic diagram of the role of RR NPs. b) Cellular uptake of RR NPs in LO2 and HepG2 cells after incubation for different time points by ICP‐MS (30 µg mL^−1^) (n = 3 independent experiments, ^****^ *p* < 0.0001, data are presented as mean ± SD). c) Flow cytometry analysis of HepG2 cells stained with DCFH‐DA after treatments with PBS, Re NPs, Ru NPs, and RR NPs in each group with or without light. d) Fluorescence microscopy images of HepG2 cells stained with DCFH‐DA after treatments with PBS, Re NPs, Ru NPs, and RR NPs in each group with or without light (Scale bar, 100 µm). e) Observation of the mitochondrial damage degree of HepG_2_ cells via monitoring the mitochondrial membrane potential after treatment with PBS, Re NPs, Ru NPs, and RR NPs in each group with or without light (Scale bar, 20 µm).

Before verifying the light‐induced ROS and CO generation properties of RR NPs (**Figure** **4**a), an in vitro cellular uptake evaluation of nanoamplifier RR NPs was conducted by using the human hepatocellular carcinoma cell line (HepG2) and the healthy liver cell line (LO2). To quantitively examine the cellular uptake kinetics, ICP‐MS was applied to detect the quantity of intracellular Re element. As shown in Figure 4b, the cellular uptake exhibited a progressive increase over a 12 h timeframe, reaching its plateau at ≈9 h. A higher cellular uptake in HepG2 compared to LO2 was observed. This increased cellular internalization by cancer cells can be attributed to enhanced electrostatic interactions between RR NPs and cancer cells, facilitated by the abundant positively charged imidazole groups of RR NPs, coupled with a more negative surface charge on cancer cells compared to normal cells.^[^ ^43^ ^]^ These factors are advantageous for RR NPs in enhancing tumor‐targeted drug delivery.

After evaluating the cellular uptake efficiencies, we conducted a quantitative intracellular assessment of ROS and CO generation capacity of nanoamplifier RR NPs. First, DCFH‐DA was utilized as an intracellular oxidant probe at a final concentration of 5 µm. Subsequently, a flow cytometry assay was conducted to detect the generation of ROS in HepG2 cells. As shown in Figure 4c, upon white light irradiation, RR NPs generated a pronounced level of ROS, slightly higher than the level observed with Ru NPs, while significantly surpassing the Re NPs by approximately two‐fold higher. This finding was further supported by fluorescent imaging analysis. As shown in Figure 4d, the RR NPs groups exhibited significant bright green fluorescence, slightly brighter than Ru NPs and much brighter than both the Re NPs and PBS control. These observed intracellular results also aligns with the extracellular results presented previously in Figure 2d.

Considering that CO has the potential to induce mitochondrial exhaustion and collapse,^[^ ^44^, ^45^, ^46^, ^47^ ^]^ we conducted mitochondrial damage analysis using a JC‐1 mitochondrial membrane potential assay kit to visualize the impact of CO on cancer cells. In healthy cells characterized by a high mitochondrial membrane potential, JC‐1 tends to form aggregates, resulting in red fluorescence. Conversely, in abnormal cells exhibiting a lower mitochondrial membrane potential, JC‐1 remains in a monomeric state, manifesting as green fluorescence.^[^ ^48^, ^49^ ^]^ In comparison to cells treated with Ru NPs and Re NPs, cells treated with RR NPs exhibited a distinct increase in green fluorescence and a noticeable decrease in red fluorescence, indicating significant mitochondrial dysfunction facilitated by CO (Figure 4e). The impact of CO was further validated by assessing cellular adenosine triphosphate (ATP) levels. As shown in Figure S23 (Supporting Information), ATP levels were significantly reduced in the RR NPs‐Light group compared to others. These findings confirmed that RR NPs possess the capability to generate ROS and release CO within tumor cells, resulting in mitochondrial dysfunction. This dual functionality is envisaged to facilitate both PDT and GT, leading to precise and significant cell apoptosis under light irradiation (Figure S24, Supporting Information).

### 2.5 In Vitro Cytotoxicity Assay of Nanoamplifiers

**Figure 5:**
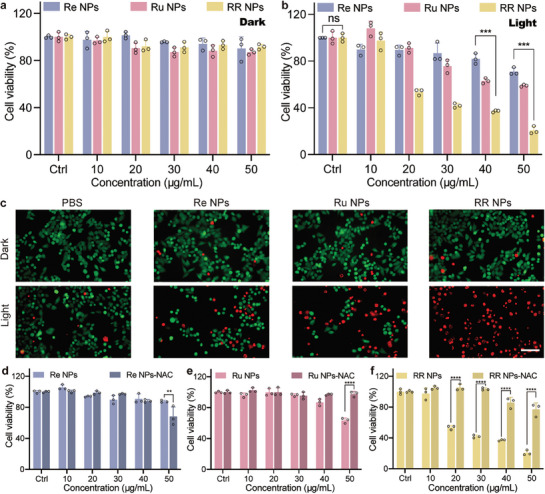
In vitro cytotoxicity assay of RR NPs. Cytotoxicity of Re NPs, Ru NPs, and RR NPs to HepG2 cells a) without and b) with light by CCK‐8 assays (n = 3 independent experiments). c) Fluorescence microscopy images of HepG2 cells co‐stained with Calcein‐AM (green) and PI (red) after being treated with PBS, Re NPs, Ru NPs, and RR NPs with and without light (Scale bars, 100 µm). HepG2 cell viability with the treatment of (d) Re NPs with and without NAC, ^**^ *p* < 0.01 (n = 3); e) Ru NPs with and without NAC, ^****^ *p* < 0.0001 (n = 3) and f) RR NPs with and without NAC, ^****^ *p* < 0.0001 (n = 3), analyzed by one‐way ANOVA, data were shown as mean ± SD.

Before further assessment of the cancer cell killing ability of nanoamplifiers, their dark toxicity was conducted on HepG2 cells with cell counting kit‐8 (CCK‐8) assay. **Figure** **5**a shows no cytotoxicity of RR NPs even up to 50 µg mL^−1^. Next, the in vitro therapeutic efficacies were scrutinized under white light irradiation. According to the CCK‐8 results, a remarkable cellular cytotoxic effect was evident at a low drug dose, 30 µg mL^−1^ for RR NPs, and yielding an approximate cell viability of 40.2% (Figure 5b). Notably, the cellular killing effect for RR NPs appears to be a 3‐fold and 2‐fold improvement in comparison to Re NPs and Ru NPs, respectively, providing strong evidence of the synergistic effect of ^1^O_2_ and CO in cancer cell killing. These findings were validated throughmethoxyl methyl ester/propidium iodide (AM/PI) assays (Figure 5c), wherein the RR NPs‐Light group displayed the most intense red fluorescence, underscoring the potent cancer cell‐killing capabilities of RR NPs.

To clarify the distinct contributions of Ru and Re‐complex in the nanoamplifier RR NPs, NAC, a ROS scavenger was introduced. Cytotoxicity studies under white irradiation revealed that, at the drug dose of 50 µg mL^−1^, the cell viability decreased for the Re NPs‐NAC group compared to the Re NPs group (Figure 5d), attributed to the increased CO release in the presence of sacrificial electron donor NAC, aligning with the results shown in Figure 2f. In contrast, the cell viability was enhanced with the addition of NAC for the Ru NPs group (Figure 5e), since NAC can neutralize the toxic ROS generated by the excited Ru‐complex. Furthermore, the cell viability was significantly enhanced in the case of RR NPs upon the addition of NAC (Figure 5f). The generation of ROS and CO were significantly suppressed in this scenario, resulting in 80% of cell viability at the dose of 50 µg mL^−1^ under light irradiation. These findings further highlighted the importance of the design of nanoamplifiers, and the indispensability of both Ru‐complex and Re‐complex in RR NPs.

### 2.6 In Vivo Biodistribution and Antitumor Efficacy Assessment of Nanoamplifiers

**Figure 6:**
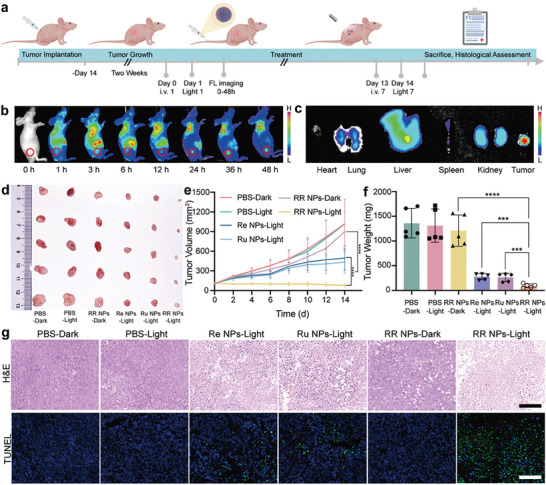
In vivo biodistribution and antitumor efficacy assessment of the RR NPs. a) Diagrammatic representation of the timeline for treatment and FL imaging in vivo. b) The in vivo biodistribution of RR NPs at 0–48 h post‐injection. The tumor site was circled. c) Fluorescence imaging of excised main organs and tumors at 48 h post‐injection. d) Images of the dissected tumors after being treated. e) Tumor growth curves during the therapeutic period (n = 5 independent experiments, ^****^ *p* < 0.0001 versus RR NPs‐Dark group, ^****^ *p* < 0.0001 versus Re NPs‐Light group). f) Tumor weight of the mice after treatment, ^****^ *p* < 0.0001 versus RR NPs‐Dark group (n = 5), ^***^ *p* < 0.001 versus the Re NPs‐Light group (n = 5), ^***^ *p* < 0.001 versus the Ru NPs‐Light group (n = 5), data were shown as mean ± SD, analyzed by two‐way ANOVA. g) H&E‐stained and TUNEL tumor sections at 15 days (Scale bars, 50 µm).

Encouraged by the excellent cancer cell‐killing capacities of RR NPs demonstrated in the in vitro experiments, we continued to investigate the biodistribution and antitumor efficacy of nanoamplifier RR NPs by using a HepG2 tumor‐bearing mouse model (**Figure** **6**a). To track the RR NPs in vivo and make them suitable for fluorescence imaging, indocyanine green (ICG) was used to modify the NPs. As shown in Figure 6b, the group of ICG‐labeled RR NPs exhibited substantial fluorescence signals at the tumor site, indicating successful tumor targeting and enrichment facilitated by the enhanced permeability and retention (EPR) effect stemming from their size. The accumulation level of RR NPs in the tumor peaked at 36 h post‐injection (Figure 6b), outperforming Free‐ICG, which only gives a weaker fluorescence signal at the tumor site (Figure S25a, Supporting Information). Quantitative analysis confirmed a higher accumulation in tumor for RR NPs (Figure 6c) than that of free ICG (Figures S25b and S26, Supporting Information). Moreover, in vivo fluorescence imaging and intensity analysis of major organs and tumor tissues at 48 h post‐injection validated the prominent tumor‐specific accumulation of RR NPs (Figure 6c).^[^ ^50^, ^51^, ^52^, ^53^ ^]^

Next, white light was applied to investigate the therapeutic benefits of PDT and CO gas therapy. As shown in Figure 6d–f, tumor volumes exhibited the most rapid growth for PBS‐Dark/Light and RR NPs‐Dark groups. In comparison, Re NPs‐Light and Ru NPs‐Light showed slightly higher tumor inhibition levels, attributed to the generated CO and ROS under light irradiation, respectively. Importantly, no tumor growth was observed for RR NPs‐Light group, the tumor volume was even decreased within a 14‐day observation period, with a tumor growth inhabitation rate of 95.6%, remarkably higher than RR NPs‐Dark (10.4%), Re NPs‐Light (72.0%), and Ru NPs‐Light groups (71.5%) (Figure 6f). These results provide evidence of the efficient suppression of tumor development under the synergistic interplay of ROS and CO. Moreover, the treatment of RR NPs significantly inhibits tumor growth of tumor‐bearing mice without inducing any discernible body weight loss (Figure S27, Supporting Information).

Consistent with these findings, tumors treated with RR NPs‐Light exhibited the most pronounced tumor cell apoptosis in the hematoxylin and eosin (H&E) staining assay and terminal deoxynucleotidyl transferase‐mediated dUTP nick‐end labeling (TUNEL) staining of tumor slices (Figure 6g). Additionally, the biosafety of RR NPs was also assessed through serum biochemical index analysis and H&E staining assay (Figures S28 and S29, Supporting Information), revealing exceptional biocompatibility properties and no apparent systemic toxicity during in vivo applications of the RR nanoamplifier.

## 3 Conclusion

In summary, we have developed biodegradable cascaded nanoamplifiers through the copolymerization of Ru‐complex (PS) and Re‐complex (CORM). Imidazole group was used as a covalent linker to introduce positive surface charges, promoting cancer cell internalization. Subsequently, flash‐nanoprecipitation enabled the self‐assembly of polymers into nanoparticles in water. The spatial proximity of PS and CORM in copolymer facilitated efficient utilization of ROS and boosted the followed‐up CO release capacity, achieving cascaded amplification for synergistic phototherapy.

Unlike conventional nanomedicines with nanocarriers or surfactants, our nanoamplifiers utilize a direct self‐assembly approach at the molecular level, ensuring a high loading of PSs and CORMs within the NPs. This design imparts “all‐into‐one” properties to the nanoamplifiers, with light‐stimulated PDT and GT offering a practical solution for “one‐for‐all” nanomedicines. Notably, their biocompatibility and biodegradability meet the requirements for potential clinical applications. This strategy reflects a commitment to advancing molecular design for optimized photophysical properties, aligning with the exigencies of prospective clinical applications in cancer therapy.

## 4 Experimental Section

### Materials

2,2′‐bipyridinyl‐4,2′‐bipyridinyl‐4,4′‐dicarboxylicacid, 1‐vinyl imidazole, RuCl_3_·xH_2_O, 2,2′‐bipyridine, silver triflate, bromopentacarbonylrhenium, and azobisisobutyronitrile were purchased from Aladin. Myohemoglobin (Mb) and 2,2,6,6‐Tetramethylpiperidoxyl (TEMPO) were bought from Sigma‐Alddrich. N‐Acetyl‐L‐cysteine (NAC), Gentian Violet, 2′,7′‐dichlorofluorescein diacetate (DCFH‐DA), Calcein‐AM/PI dual‐staining kit, and Cell Counting Kit‐8 (CCK‐8) were obtained from Beyotime Biotechnology. Fetal bovine serum (FBS) and Dulbecco's Modified Eagle Medium (DMEM) were purchased from Hyclone (USA).

All the other solvents including N, N‐dimethylformamide (DMF), methanol, ethanol, acetic acid, trifluoroacetic acid, and paraformaldehyde were purchased from Sinopharm Group Co. Ltd.HepG2 cells and LO2 cells were provided by the Institute of Biochemistry and Cell Biology (Shanghai, China). Nude mice were obtained from Shanghai SLAC Laboratory Animal Co. Ltd (Shanghai, China).

### Polymer Synthesis and Characterizations

*General synthetic processor for homo‐polymerization* (Scheme S4, Supporting Information): Re‐complex or Ru‐complex (1 eq) and azobisisobutyronitrile (AIBN) (1.4 eq) were added to a 25 mL Schlenk tube with dry tetrahydrofuran (THF): acetonitrile (ACN): Ethanol (1:2:3). The tube was cooled in liquid nitrogen, evacuated and purged with dry nitrogen for three times, stirred at 80 °C for 72 h. After cooling to ambient temperature, the solvent was removed under reduced pressure by rota evaporate. Diethyl ether (Et_2_O, ≈30 mL) was added to the solution, the resulting precipitate was filtered, washed with diethyl ether and THF, centrifuged, washed with ethanol three times, then the precipitation was dissolved in ethanol (1 mL) and water (1 mL), after being treated with sonication, saturated potassium hexafluorophosphate (KPF6) aqueous solution was added dropwise until no precipitation was formed. The polymer was filtered and add ethanol (10 mL), and centrifuged, this processor was continuing until AIBN removed washed with water (10 mL), dried under vacuum.

*Synthesis of RR polymer* (Scheme S4, Supporting Information): Re‐complex (1 eq), Ru‐complex (1 eq), and azobisisobutyronitrile (AIBN) (1.4 eq) were added to a 25 mL Schlenk tube with dry THF: CAN: Ethanol (1:2:3). The tube was cooled in liquid nitrogen, evacuated and purged with dry nitrogen for three times, stirred at 80 °C for 72 h. After cooling to ambient temperature, the solvent was removed under reduced pressure by rota evaporate. Et_2_O was added to the solution, the resulting precipitate was filtered, washed with diethyl ether and THF, centrifuged, washed with ethanol three times, then the precipitation was dissolved in ethanol (1 mL) and water (1 mL), after being treated with sonication, saturated KPF_6_ aqueous solution was added dropwise until no precipitation was formed. The polymer was filtered and add ethanol (10 mL), centrifuged, this processor was continuing until AIBN removed washed with water (10 mL), dried under vacuum. Red solid (25%).

### Characterizations of Re Polymer, Ru Polymer, and RR Polymer

^1^H NMR spectral data of Re Polymer, Ru Polymer, and RR Polymer were obtained on a 600 MHz NMR spectrometer (^1^H‐NMR, Bruker AV600, Switzerland). Elemental analysis of RR polymer was performed by ICP‐MS (NexION2000, PerkinElmer). The UV–vis absorption spectrum was measured using a spectrometer (UV‐2550, Shimadzu, Japan). The redox potential of Re Polymer and RR polymer were carried out by using cyclic voltammograms recorded in a standard three‐electrode cell using an Autolab potentiostat (PGSTAT302) controlled with GPES software. The fluorescence spectrum was measured using a spectrometer (Hitachi, Model F‐7100, Japan). The concentrations of different groups were shown below: Ru NPs (30 µmol mL^−1^), Ru (30 µmol mL^−1^) + Re (30 µmol mL^−1^) NPs, and RR NPs (60 µmol L^−1^, according to the repeat units of Re‐complex and Ru‐complex).

### Preparation of Re/Ru/RR NPs and Re Polymer + Ru Polymer Blended NPs

Preparation of nanoparticles using microfluidic devices. In short, the acetonitrile solution of polymer (Re/Ru/RR polymer) and aqueous solution was injected into the microfluidic chip through a syringe, and nanoparticles of different sizes were prepared by controlling different flow rates. The prepared solution was then placed in a fume hood to evaporate, and the final nanoparticles are obtained.

### Characterizations of Re/Ru/RR NPs and Re Polymer + Ru Polymer Blended NPs

Hydrodynamic diameters and zeta potentials of Re/Ru/RR NPs were measured by dynamic light scattering (DLS, Zetasizer Nano ZS‐90, Malvern, UK). The morphology of the as‐synthesized nanoparticles was observed using transmission electron microscopy (TEM, JEM‐2100, JEOL, Japan). The UV–vis absorption spectrum was measured using a spectrometer (UV‐2550, Shimadzu, Japan). Elemental analysis was performed using a field emission transmission electron microscope F200 (Mapping, JEOL JEM‐F200). Time resolved single photon counting was measured using a transient spectrometer (Edinburgh FLS1000, Edinburgh, England), 485 nm was used for the excitation, 625 nm was used for emission.

### Hemolysis Test

500 µL of blood was taken from the mouse eyeballs, centrifuged at 4 °C and 3500 rpm for 5 min, the supernatant was carefully discarded, and then washed with phosphate‐buffered saline (PBS) until the supernatant was colorless. Then dilute 20 times with 10 mL of PBS. Positive control: 200 µL red blood cell suspension + 800 µL H_2_O. Negative control: 200 µL red blood cell suspension + 800 µL PBS. Experimental group: 200 µL red blood cell suspension + 800 µL CPPDH of different concentrations (dissolved in PBS). Each group was incubated at 37 °C for 6 h and then centrifuged at 4 °C and 3500 rpm for 5 min. 100 µL of the supernatant was transferred to a 96‐well plate, and the optical density (OD) value at 562 nm was measured. The calculation formula for the hemolysis rate is: (OD value of sample‐negative OD value)/(positive OD value‐negative OD value) * 100%. Hemolysis rate >5% can be regarded as hemolysis.

### Photodegradation Experiment

Place the RR NP in the eppendorf tube, then illuminate it with light‐emitting diodes (LED), and take samples with different illumination times for UV, DLS, and TEM characterization.

### In Vitro Detection of ROS Generation

The ROS generation of Ru/RR NP was evaluated by a 2,7‐dichlorodihydrofluorescein diacetate (DCFH‐DA) kit. The DCFH‐DA probe was pretreated with sodium hydroxide (10 mmol) over 12 h to obtain DCFH and diluted with double distilled water. When oxidized by ROS, DCF will emit fluorescence. Then Ru/RR NP at a concentration of 25 µg mL^−1^ [Ru] mixed with an activated probe were exposed to light for 5 min. Samples were taken and measured in minutes. The fluorescence intensity of DCF oxidized by ROS was counted by a fluorescence spectrophotometer (Hitachi F‐2500, Japan) (Ex: 488 nm, Em: 525 nm). The groups without light were the controls.

Another method has also been used to detect the production of ROS from RR NP. 2,2,6,6‐Tetramethyl‐4‐piperidone monohydrate (TEMP) was used as the trapping reagent to identify the ^1^O_2_ radicals by the ESR spin‐trap technique. (Bruker A300, Germany) RR NP (50 µg mL^−1^), and TEMP (50 mM) were added into 2 mL of double distilled water. All samples were exposed to light for 5 min and ESR spectra were then recorded. The groups without light were the controls.

### In Vitro Detection of CO Release

Myohemoglobin (Mb) (10 mg) was dissolved in phosphate buffer saline (PBS) (10 mL) (0.1 m, pH 7.4). Add 0.1% sodium dithionite, fill with nitrogen, and exclude air, continue for 30 min to obtain Deoxy‐Mb. Then the Re/RR NPs were mixed with the above deoxy‐Mb by light for 5 min. Samples were taken and measured in minutes. The absorption curve (500–600 nm) was recorded by an ultraviolet absorption spectrometer. NAC, a free radical capture squeeze, was applied to remove the effects of ROS. The experimental group that removed ROS kept the concentration of NAC at 5 mm.

Gas chromatography was also used to detect the CO production of RR NPs. The experiment was performed in 3 mL gastight vials with argon (Ar) for 30 min; then, 1 mL of Re/RR NPs aqueous solution (25 µg ml^−1^ of [Re]) was added into the above gastight vials. An LED PAR38 lamp (17 W, 5000K, Zenaro Lighting GmbH, λ > 420 nm) was used as the light source. Take samples for analysis after every 1 min of light exposure. The generated CO was quantified by a gas chromatograph system (GC9790 Plus, Fuli Instruments) using Ar as carrier gas.

### Cell Culture

The LO2 cells and HepG2 cells were incubated in a cell culture medium (DMEM, FBS 10% (v/v), penicillin/streptomycin 100 µg mL^−1^) at a suitable growing environment (5% CO_2_, 37 °C).

### In Vitro Cytotoxicity Study

LO2 cells and HepG2 cells were seeded in 96‐well plates for 12 h, respectively, with a density of 1 × 10^4^ cells/well. Then the medium was replaced by different concentrations of Re/Ru/RR NPs and in DMEM with FBS for 12 h. Next, after being washed with PBS, every well was filled with DMEM. Immediately, the experimental group was exposed to light for 5 min and the groups without light were as the controls. After 12 h, the cytotoxicity was tested by CCK‐8.

To verify the role of ROS in this system, NAC was also used in the culture medium to determine the lethality of different nanoparticles to cells. HepG2 cells were cultured in 96‐well plates (1 × 10^4^ cells/well) for 12 h. The medium was removed with substitutes of Re/Ru/RR NPs containing NAC (5 mm, pH 7.4) for 12 h. Then the plates were washed and added to medium containing NAC (5 mm, pH 7.4). The light groups suffered light and another 12 h incubation. The cell viability was surveyed with CCK‐8.

### In Vitro Cell Uptake and Targeting

LO2 cells and HepG2 cells were seeded in 6‐well plates for 24 h, respectively, with a density of 1 × 10^6^ cells/well. Then the medium was replaced by RR NPs (30 µg mL^−1^) in DMEM for 0, 1, 3, 6, 9, and 12 h. The cells were collected at different time points after multiple washes. The amount of cellular uptake was investigated by ICP‐MS (NexION2000, PerkinElmer).

### In Vitro ROS Generation in Cell

The DCFH‐DA probe was chosen for cellular ROS detection by Fluorescence Inverted microscope and flow cytometry. HepG2 cells were incubated in 12‐well plates (4 × 10^4^ cells/well) for 12 h. And Re NPs and RR NPs at a concentration of 25 µg mL^−1^ [Ru] medium were added to cell plates for 9 h culture. Then, light groups were exposed to light for 5 min. And plates were washed with PBS to remove the extracellular materials and filled with DCFH‐DA in PBS for 30 min.

After being imaged with a fluorescence‐inverted microscope, the cells were collected for a flow cytometry test. (ATTUNE 22 NXT, Thermo Fisher Scientific, USA)

### In Vitro Assessment of CO Cytotoxicity

The HepG2 cells were seeded onto confocal special purpose 6‐well culture plates with 80 000 cells per well. Then 12 h later, Re/Ru/RR NPs solution was added. After another 9 h, the cells were washed twice with PBS. The fresh medium was added to the culture plate, and each well in the light group was exposed to light for 5 min. Fresh medium containing JC‐1 was added to the culture plates and imaged by CLSM (Zeiss LSM 510 Meta, Germany).

### In Vitro Detection of Intracellular ATP Levels

The HepG2 cells were seeded onto 6‐well plates at a density of ≈200 000 cells per well. The cells were divided into eight experimental groups for the comparative study: PBS group, Re NPs‐Dark group, Ru NPs‐Dark group, RR NPs‐Dark group, PBS‐Light group, Re NPs‐Light group, Ru NPs‐Light group, and RR NPs‐Light group. After 24 h of incubation, the medium was replaced by fresh medium containing Re NPs, Ru NPs, and RR NPs at concentration of 50 µg mL^−1^, respectively. Following an additional 12 h of incubation, the cells were washed twice with PBS and the medium was refreshed. Subsequently, the PBS‐Light group, the Re NPs‐Light group, the Ru NPs‐Light group, the RR NPs‐Light group were irradiated with LED Light for 5 min and then incubated for another 12 h. The supernatant was collected post‐incubation by lysing the cells and then added to the ATP assay kit. The Luminescence intensity, indicating the ATP levels, was measured using a Spectra Max iD3 multifunctional plate reader.

### In Vitro Apoptosis Assay

The HepG2 cells were seeded onto 6‐well plates at a density of ≈200 000 cells per well. The cells were divided into eight experimental groups for the comparative study: PBS group, Re NPs ‐Dark group, Ru NPs‐Dark group, RR NPs‐Dark group, PBS‐Light group, Re NPs‐Light group, Ru NPs‐Light group, and RR NPs‐Light group. After 24 h of incubation, the medium was replaced by fresh medium containing Re NPs, Ru NPs, and RR NPs at a concentration of 30 µg mL^−1^. Following an additional 12 h of incubation, the cells were washed twice with PBS and the medium was refreshed. Subsequently, the PBS‐Light group, the Re NPs‐Light group, the Ru NPs‐Light group, the RR NPs‐Light group were irradiated with LED Light for 5 min and then incubated for another 12 h. The cells were collected and stained with the Annexin V‐FITC for and apoptosis assay. Quantitative fluorescence intensity was measured by flow cytometry to determine the levels of apoptosis.

### Animal Model Establishment

All animal study protocols were consistent with the principles and procedures, which defined in the Guide for the Care and Use of Laboratory Animals, approved by Institutional Animal Care and Use Committee of Xiamen University (NO. XMULAC20190001). The Balb/c nude mice (male, 5–6 weeks, 20 ± 2 g) were provided by Xiamen University. The subcutaneous HCC tumor model of Balb/c nude was built by inoculation of thick HepG2 cells suspension (2 × 10^6^ cells/0.1 ml) on the right thigh. When modeling was successful, these models were applied for the follow‐up animal experiences. The tumor volume was estimated by the formula: V = L × D^2^/2, in which L and D were the longest and the shortest diameter of the tumor, respectively.

### Fluorescence Imaging

To evaluate the in vivo distribution of RR NP, HepG2 tumor‐bearing mice were randomly assigned to two groups: 1) Free ICG; and 2) ICG‐labeled RR NP. After intravenous injection of corresponding ICG at 0.5 mg kg^−1^, the in vivo fluorescence distribution was observed using an in vivo imaging system (Living Image) (λ_ex_ = 740 nm, λ_em_ = 820 nm). At 48 h after injection, the mice were sacrificed, and their tumor tissues and main organs (heart, liver, spleen, lung, and kidneys) were excised for FL imaging ex vivo.

### In Vivo Antitumor Efficiency Evaluation

HepG2 subcutaneous tumor‐bearing mice were randomly divided into 6 groups (n = 5): 1) PBS; 2) PBS‐Light; 3) Re NPs‐Light; 4) Ru NPs‐Light; 5) RR NPs‐Dark; 6) RR NPs‐Light. They were administered by tail vein injection at a dose of 5 mg kg^−1^. 24 h after injection, the corresponding mice were exposed to light for 5 min. A total of 7 administrations and 7 irradiations were performed. The tumor volumes and body weights of mice were monitored every 2 days over 14 days. At the end of treatment, all mice were sacrificed and the tumors as well as the major organs were collected, photographed, weighed, and then fixed in 4% paraformaldehyde for hematoxylin and eosin (H&E) staining and terminal deoxynucleotidyl transferase‐mediated dUTP‐biotin nick end labeling assay (TUNEL) staining. The tumor growth inhibition rate (TGIR) was calculated according to the following equation: TGIR (%) = (WC − WT)/WC × 100%, where WC was the tumor weight of the PBS group and WT represented the tumor weight of mice after the treatments.

### In Vivo Biocompatibility Assessment

The healthy Balb/c nude mice were administrated systematically with PBS, Re NP, Ru NP, and RR NP (5 mg kg^−1^) on the first day and third day, respectively. After 1 week, blood samples were taken for blood cell count (WBC, RBC, PLT, HGB) and serum biochemical assessment (indicators of liver function: Alanine aminotransferase (ALT), Aspartate aminotransferase (AST); indicators of kidney function: Creatinine (CREA).

### Statistical Analysis

Except for the in vivo experiments which were repeated five times, all other experiments were repeated three times. For comparisons of more than two groups, the Brown‐Forsythe test was used to assess similar variances, followed by one‐way ANOVA or two‐way ANOVA with Tukey's multiple comparisons test. Statistical analysis of the data was carried out using GraphPad Prism software 9 and data were shown as mean ± SD. Sample size (n) for each statistical analysis is indicated in each figure legend. ^*^ *p* < 0.05, ^**^ *p* < 0.01, ^***^ *p* < 0.001, and ^****^ *p* < 0.0001 which was considered significant, moderately significant, highly significant, and much more highly significant respectively.

## Conflict of Interest

The authors declare no conflict of interest.

## Supporting information

Supporting Information

## Data Availability

The data that support the findings of this study are available from the corresponding author upon reasonable request.
