# Supplementary material for: Biodegradable Ruthenium‐Rhenium Complexes Containing Nanoamplifiers: Triggering ROS‐Induced CO Release for Synergistic Cancer Treatment
Source: Adv Sci (Weinh). 2024 Jul 12;11(35):2403795. doi: 10.1002/advs.202403795 (PMC11425273; doi:10.1002/advs.202403795)
Supplement: Supplementary file 1 — Supporting Information [file ADVS-11-2403795-s001.docx]

**Supporting Information**

for

**Biodegradable Ruthenium-Rhenium Complexes Containing Nanoamplifiers: Triggering ROS-Induced CO Release for Synergistic Cancer Treatment**

Aijie Liu^1,2†*^, Zhenkun Huang^1†^, Xiangfu Du^1^, Naresh Duvva^3^, Yuting Du^1^, Zihao Teng^1^, Zhihuan Liao^1^, Chen Liu^1^, Haining Tian^3*^, Shuaidong Huo^1*^

1 State Key Laboratory of Cellular Stress Biology, Fujian Provincial Key Laboratory of Innovative Drug Target Research, School of Pharmaceutical Sciences, Xiamen University, Xiamen 361102, China.

2 Shenzhen Research Institute of Xiamen University, Shenzhen, 518057, China

3 Department of Chemistry-Ångström Lab, Box 523, SE 751 20, Uppsala University, Sweden.

† These authors contributed equally to this work.

*Correspondence to: huosd@xmu.edu.cn, haining.tian@kemi.uu.se, and aijieliu@xmu.edu.cnu

**Scheme S1.** Synthesis of compound (4).

**1. Synthesis of compound (4)**

**Step (ⅰ)**: 2.5 g of 2,2'-bipyridinyl-4,2'-bipyridinyl-4,4'-dicarboxylic acid and 60ml of absolute ethanol in 50 g of conc. H_2_SO_4_ at 85 °C, for 18 hours., which was then cooled by pouring on ice cold water, which was further neutralized with 25 % aq. NaOH and the precipitate were filtered out and thoroughly washed with deionized water which was used without further purification after drying overnight (84 %). ^1^H NMR (400 MHz, CDCl_3_): d = 8.97 (s, 2 H), 8.89 (d, J = 4.9 Hz, 2 H), 7.94 (dd, J = 5.0, 1.6 Hz, 2 H), 4.48 (q, J = 7.1 Hz, 4 H), 1.47 (t, J = 9.2, 5.0 Hz, 6 H).

**Step (ⅱ)**: Compound 1 (750 mg, 2.4 mmol) was suspended in ethanol (50 mL) followed by the addition of sodium borohydride (2 g, 53 mmol). The mixture was heated to reflux at 65 ^o^C under argon for 6 h. The formation of a gel on the surface of the reaction mixture was observed after approximately 1 h. A further 25 mL of ethanol was added to dissolve the gel. After cooling to room temperature, saturated NH_4_Cl (aq) (100 mL) was added to the mixture, which was stirred for 15 min. The ethanol was removed under vacuum and the resulting white precipitate was dissolved in the minimum quantity of water (ca. 150 mL). The solution was extracted with ethyl acetate (5 V 50 mL) and the combined organic fractions were dried with MgSO_4_. The solvent was removed under vacuum to give a pale pink solid. Yield: 306 mg (50 %).

**Step (ⅲ)**: Compound 2 (674 mg, 3.12 mmol) was dissolved in 48 % HBr (40 mL) and 98 % H_2_SO_4_ (14 mL). The resulting orange fuming solution was heated to reflux at 100 ^o^C for 18 h. Upon cooling to room temperature, the mixture was neutralized with saturated NaOH (aq) (ca. 200 mL). The resulting white precipitate was filtered off with a frit and washed with water (300 mL) and air-dried. The solid was then dissolved in chloroform (40 mL), dried over MgSO_4_ and filtered. The solvent was removed under vacuum leaving a white solid. Yield 842 mg (79 %).

**Step (ⅳ)**: Compound 3 (0.342 g, 1 mmol) in 30 ml of freshly distilled dry toluene was added dropwise into the stirring toluene solution of 1-vinyl imidazole (0.377 g, 4 mmol) under an argon atmosphere. The reaction mixture was refluxed for 72 h. After cooling to room temperature, the toluene solvent was evaporated in vacuo and the lower viscous liquid was washed two times with dry THF (2 × 30 mL) and ethyl ether (2 × 100 mL), respectively. The solvent was removed and dried under a vacuum overnight to obtain the compound 4 as light pink solid. Yield: 82%. ^1^H NMR (400 MHz, D_2_O) δ 9.12 (s, 1H), 8.56-8.53 (d, J = 5.2 Hz, 1H), 7.94 (s, 1H), 7.75-7.73 (d, J = 1.9 Hz, 1H), 7.53-7.51 (d, J = 1.9 Hz, 1H), 7.37-7.33 (d, J = 3.8 Hz, 1H), 7.05 (dd, J = 15.6, 8.7 Hz, 1H), 5.75-5.67 (dd, J = 8.5, 3.2 Hz, 1H), 5.53 (s, 2H), 5.34 (dd, J = 8.7, 2.9 Hz, 1H).

**Scheme S2.** Synthesis of compound (6).

**2. Synthesis of compound (6)**

**Step (i)**: 1.56 g (5.96 mmol) RuCl_3_·3H_2_O was dissolved in 10 ml DMF, and then 1.68 g (0.4 mmol) LiCl and 1.87 g (12 mmol) 2,2′-bipyridine were added. The mixture was refluxed at 145 °C under the protection of N_2_ for 8 hours and then cooled to room temperature. 50 ml acetone was added to the reaction mixture and froze at 0 °C overnight. Purple black precipitates were obtained by filtration, which were washed with small amounts of cooled water, and then washed successively with 5 ml methanol and 20 ml ethyl ether. The final product of Ru(bpy)_2_Cl_2_·2H_2_O (Compound 5) was obtained after drying in a vacuum. Yield: 43%. ^1^H NMR (400 MHz, DMSO-d6, 25 °C): δ = 9.96 (d, 2H), 8.85 (d, 2H), 8.50 (d, 2H), 8.08 (t, 2d), 7.78 (t, 2H), 7.68 (t, 2H), 7.50 (d, 2H), 7.12 (t, 2H).

**Step (ii)**: A mixture of ruthenium (II) precursor complex (298 mg, 0.572 mmol) and silver triflate (312 mg, 1.21 mmol) in acetonitrile (100 mL) was degassed and then heated at reflux in the dark for 32 h. After cooling to room temperature, the resulting precipitate was filtered and rinsed with acetonitrile (5 mL). The solvent of the orange filtrate was removed under reduced pressure, and compound (6) was obtained as an orange solid (80%). ^1^H NMR (400 MHz, CD_3_CN): δ (ppm) = 9.32 (ddd, J = 5.6, 1.5, 0.8 Hz, 2H), 8.52 (dt, J = 8.2, 1.0 Hz, 2H), 8.41-8.34 (m, 2H), 8.27 (ddd, J = 8.2, 7.7, 1.5 Hz, 2H), 7.94 (ddd, J = 8.2, 7.6, 1.5 Hz, 2H), 7.85 (ddd, J = 7.7, 5.6, 1.3 Hz, 2H), 7.59 (ddd, J = 5.7, 1.5, 0.8 Hz, 2H), 7.25 (ddd, J = 7.6, 5.7, 1.3 Hz, 2H), 2.27 (s, 6H).

**Scheme S3.** Synthesis of Re-Complex and Ru-Complex.

**3. Synthesis of Re-complex and Ru-complex**

**Re-complex (Scheme S3):** To a Schlenk flask added under the nitrogen atmosphere, a mixture of compound 4 (0.1 g, 0.189 mmol) and Re(CO)_5_Br (0.084 g, 0.21 mmole) in a 15 ml of freshly distilled dry toluene was added. The reaction mixture was continued to stir for 3 days at 110 ˚C under nitrogen condition. After that, the reaction mixture was cooled to room temperature and removed the solvent by rota evaporated. Add 15ml of ethyl ether then the solid was filtered off and thoroughly washed with THF and dried under vacuum overnight to obtain the compound Re-Complex as yellow solid. Yield: 68%.

**Ru-complex (Scheme S3)**: A suspension of precursor compound (6) (50 mg, 0.1 mmol), the compound (4) (66.16 mg, 0.124 mmol) and silver triflate (60 mg, 0.192 mmol) in ethylene glycol (8 mL) was degassed for 30 min and then heated at 120 °C for 4 days and cool to room temperature. Puriﬁcation was carried out as outlined below. Puriﬁcation: The mixture was taken up in methanol and acetone and filtered through a pad of celite, which was then rinsed with acetone. The solvent was removed under reduced pressure and the residue was purified by column chromatography (SiO_2_, acetone, water, saturated aqueous KNO_3_ 100:10:1 → acetone, water, saturated aqueous KNO_3_ 100:50:10). Saturated aqueous KPF_6_ solution was added to the second red-colored fraction which contained the desired triad. The organic solvent was removed under reduced pressure. The resulting precipitate was filtered and washed with cold water and Et_2_O. The red solid was taken up in CH_2_Cl_2_ (50 mL) and the organic phase was washed with water containing 10% acetate buffer (pH 5, 0.1 M, 10 mL) (3 × 50 mL). The combined aqueous phases were extracted with CH_2_Cl_2_ (1 × 10 mL). To the combined aqueous phases was added saturated aqueous KPF_6_ solution and the mixture was stored for 0.5 h at 5 °C. Filtration and washing with cold water (30 mL) and Et_2_O (30 mL) yielded a red solid, that was collected from the frit with acetone and subjected to column chromatography (SiO_2_, acetone → acetone, water, saturated aqueous KNO_3_ 100:10:1 → acetone, water, saturated aqueous KNO_3_ 100:50:10). Saturated aqueous KPF_6_ solution and acetate buffer (pH 5, 0.1 M) was added to the second red fraction which contained the desired triad. The organic solvent was removed under reduced pressure. The resulting precipitate was filtered and washed with cold water and Et_2_O. The red solid was collected from the frit with acetone. Solvent removal under reduced pressure yielded Ru-complex as a red solid (40%).

**Scheme S4.** Synthesis of Re Polymer, Ru Polymer and RR Polymer.


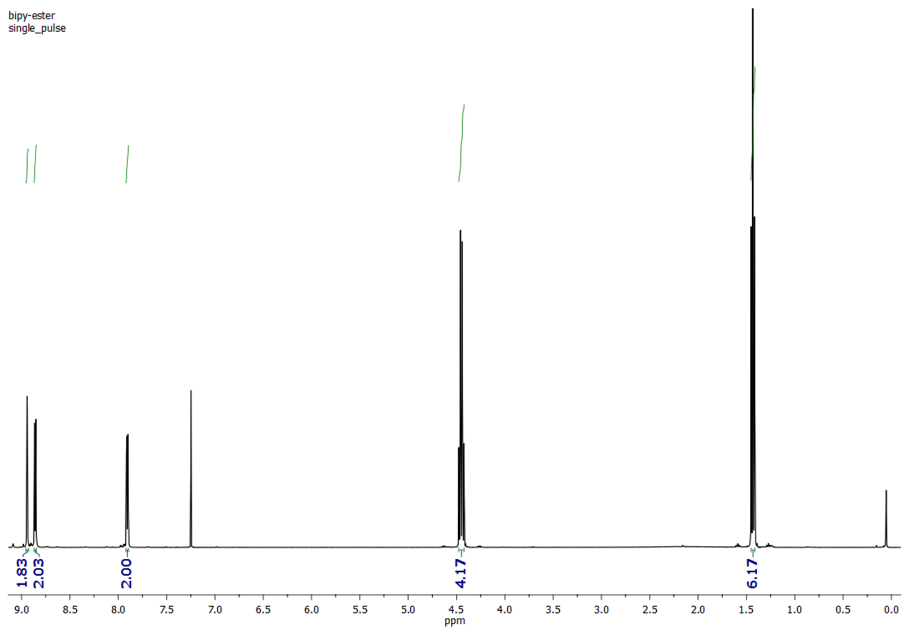


Figure S1. ^1^H NMR spectra of (1).


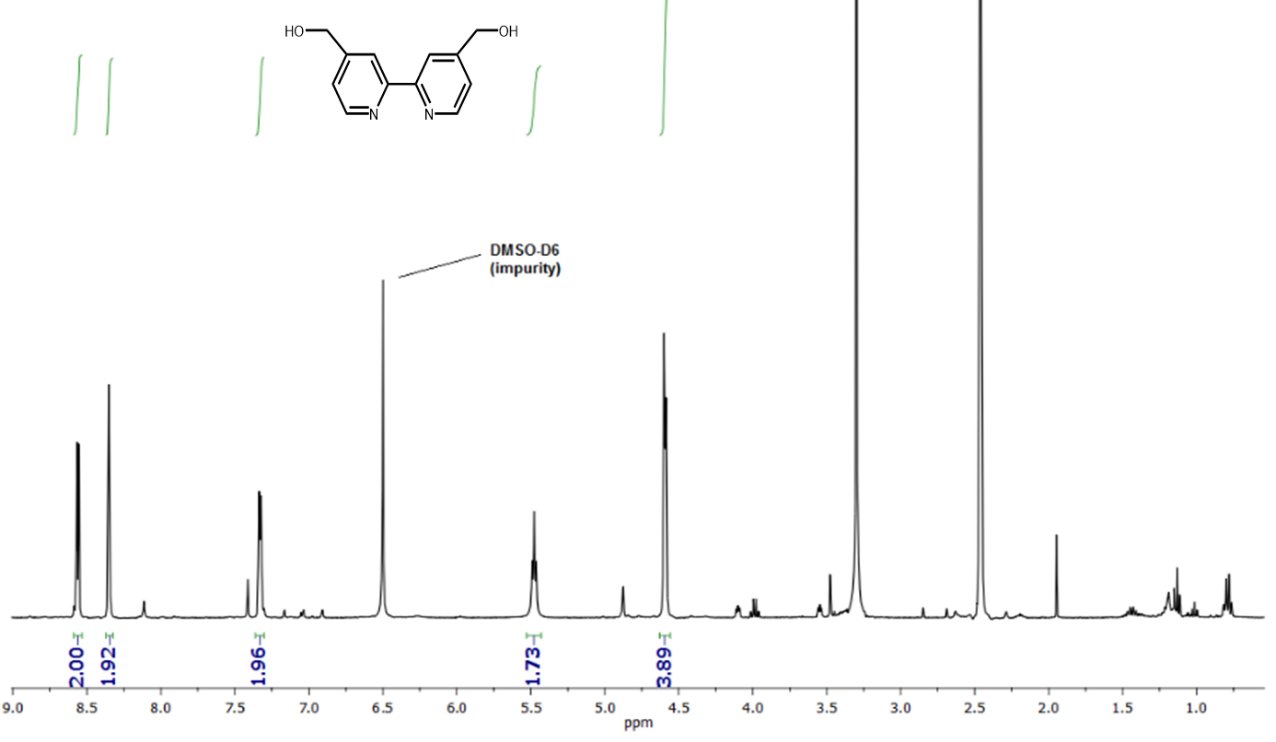


Figure S2. ^1^H NMR spectra of (2).


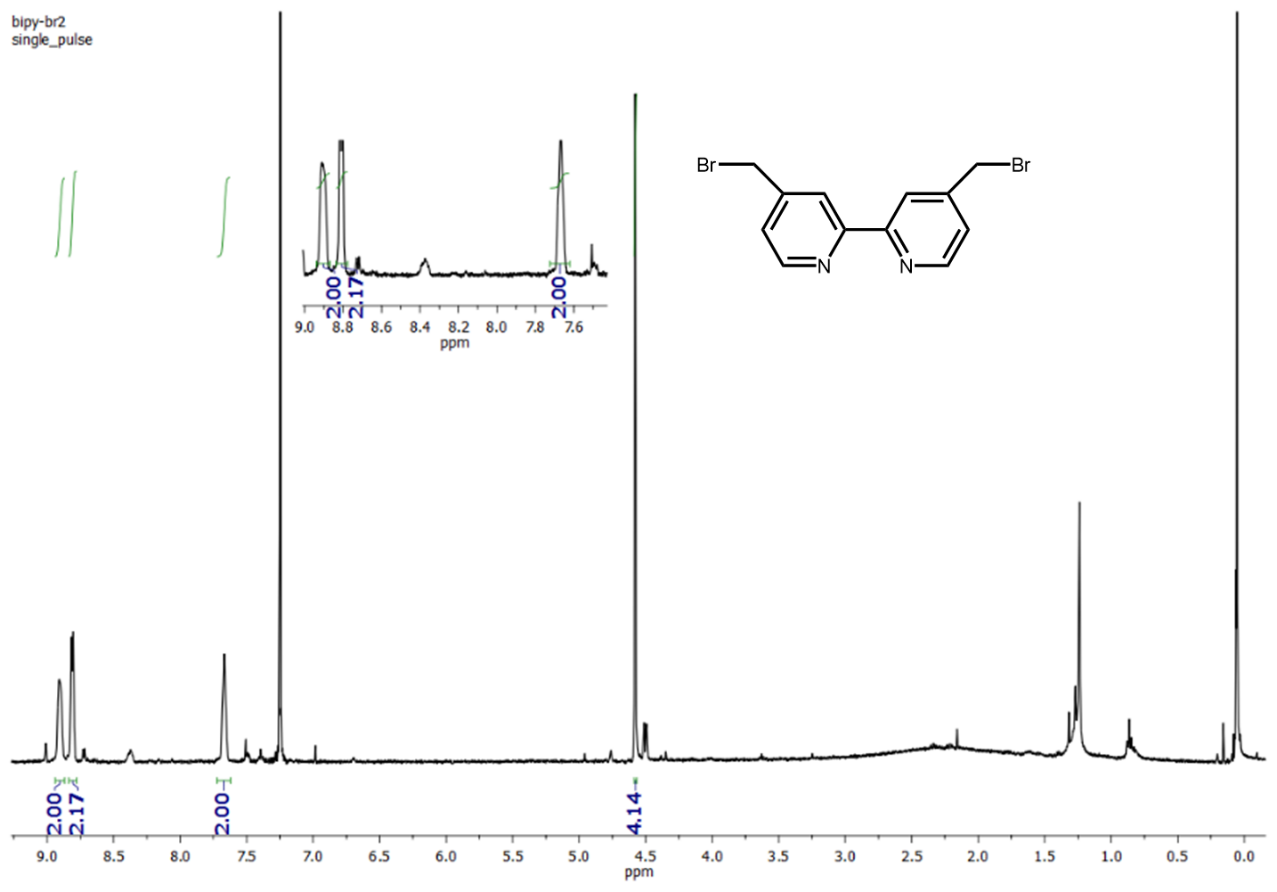


Figure S3. ^1^H NMR spectra of (3).


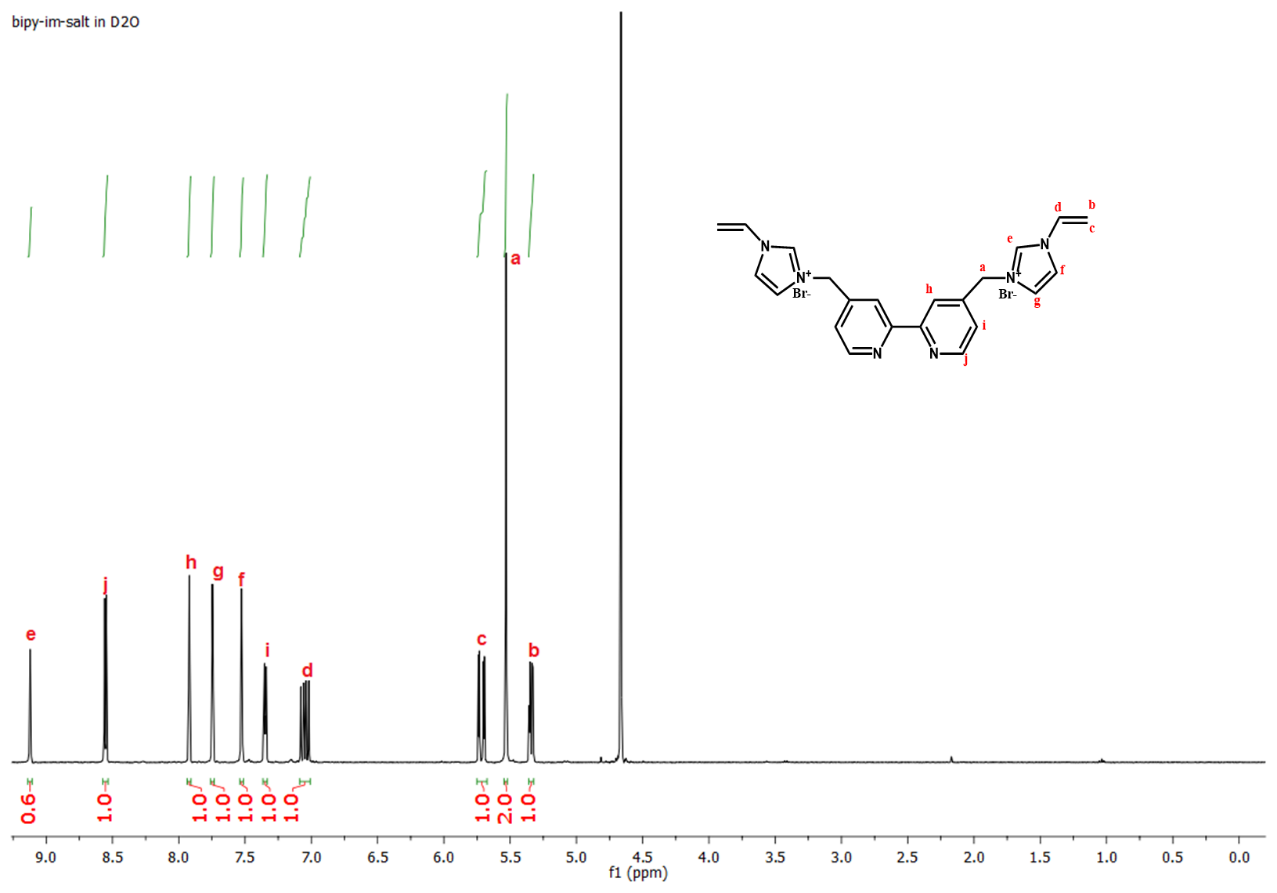


Figure S4. ^1^H NMR spectra of (4).


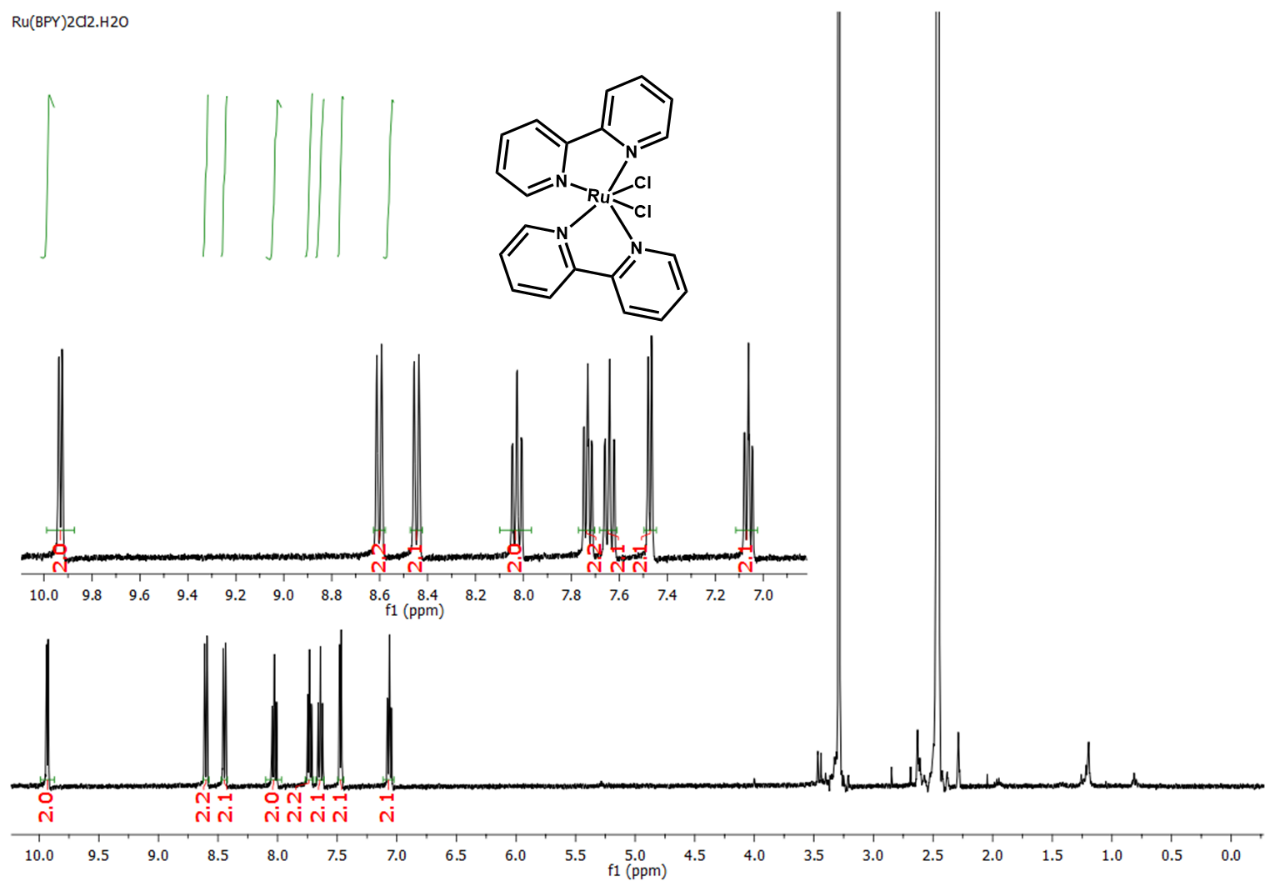


Figure S5. ^1^H NMR spectra of (5).


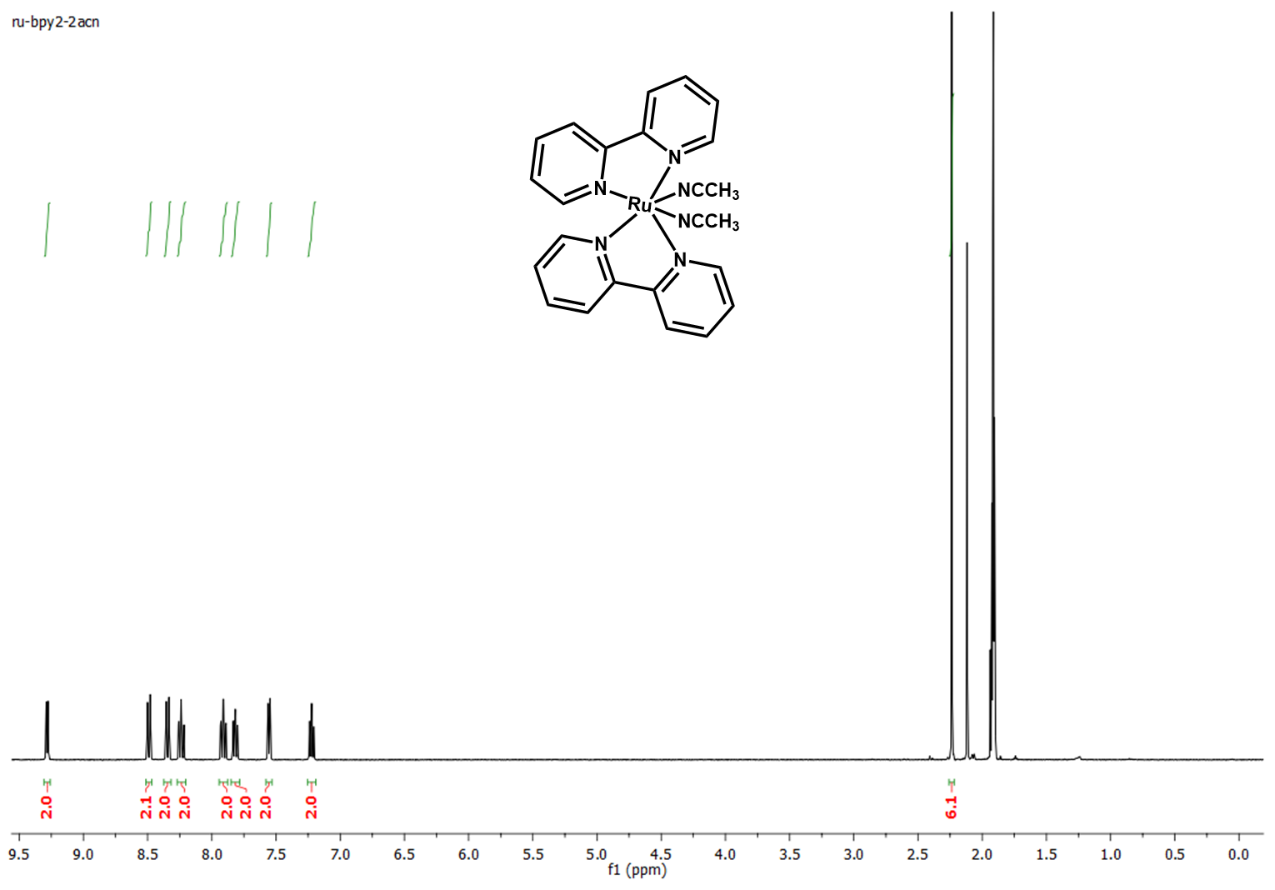


Figure S6. ^1^H NMR spectra of (6).


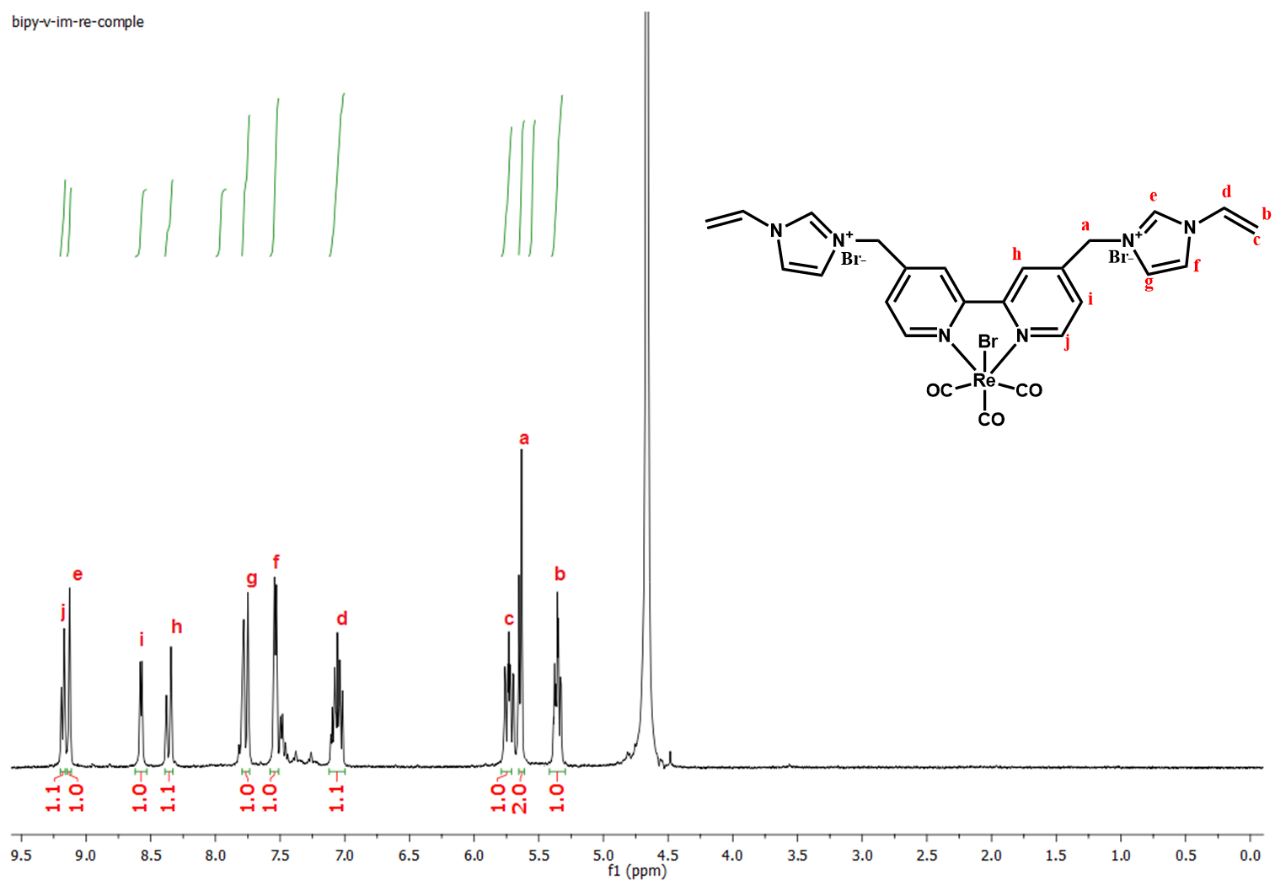


Figure S7. ^1^H NMR spectra of Re-Complex.


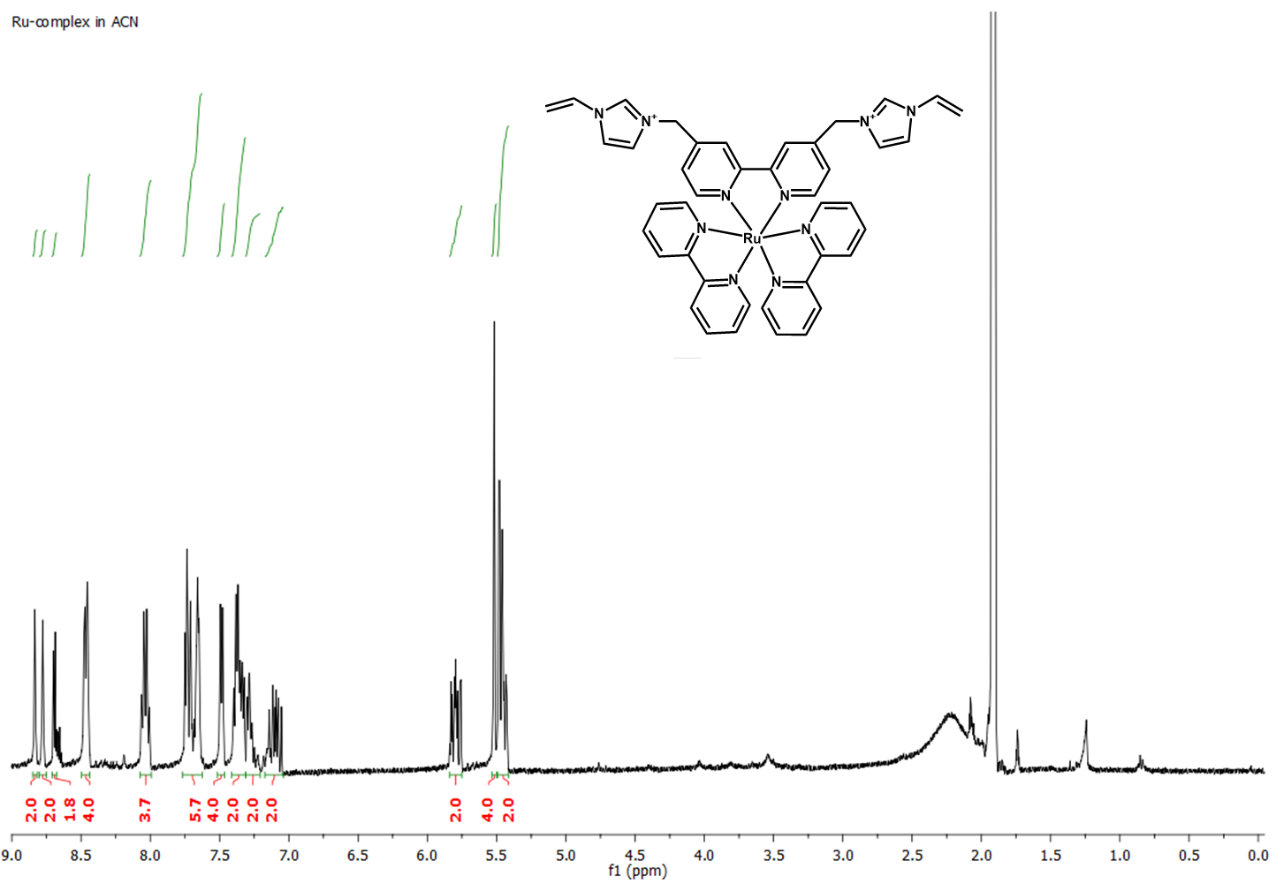


Figure S8. ^1^H NMR spectra of Ru-Complex.


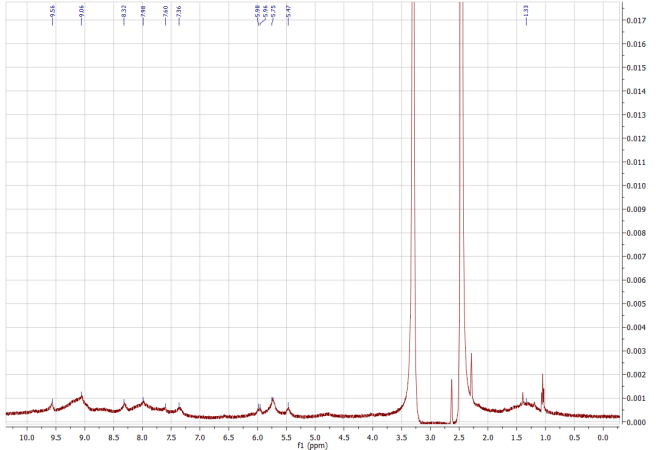


Figure S9. ^1^H NMR spectra of Re Polymer.


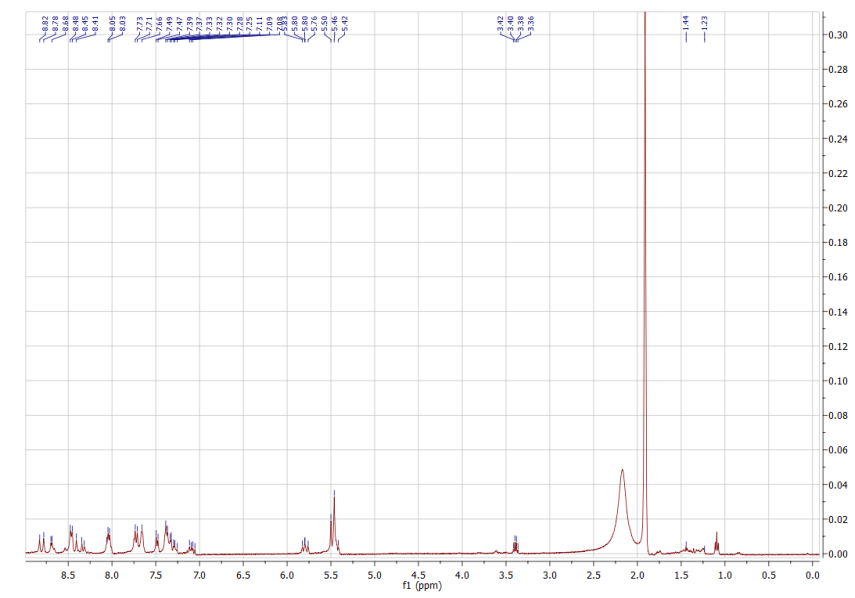


Figure S10. ^1^H NMR spectra of Ru Polymer.


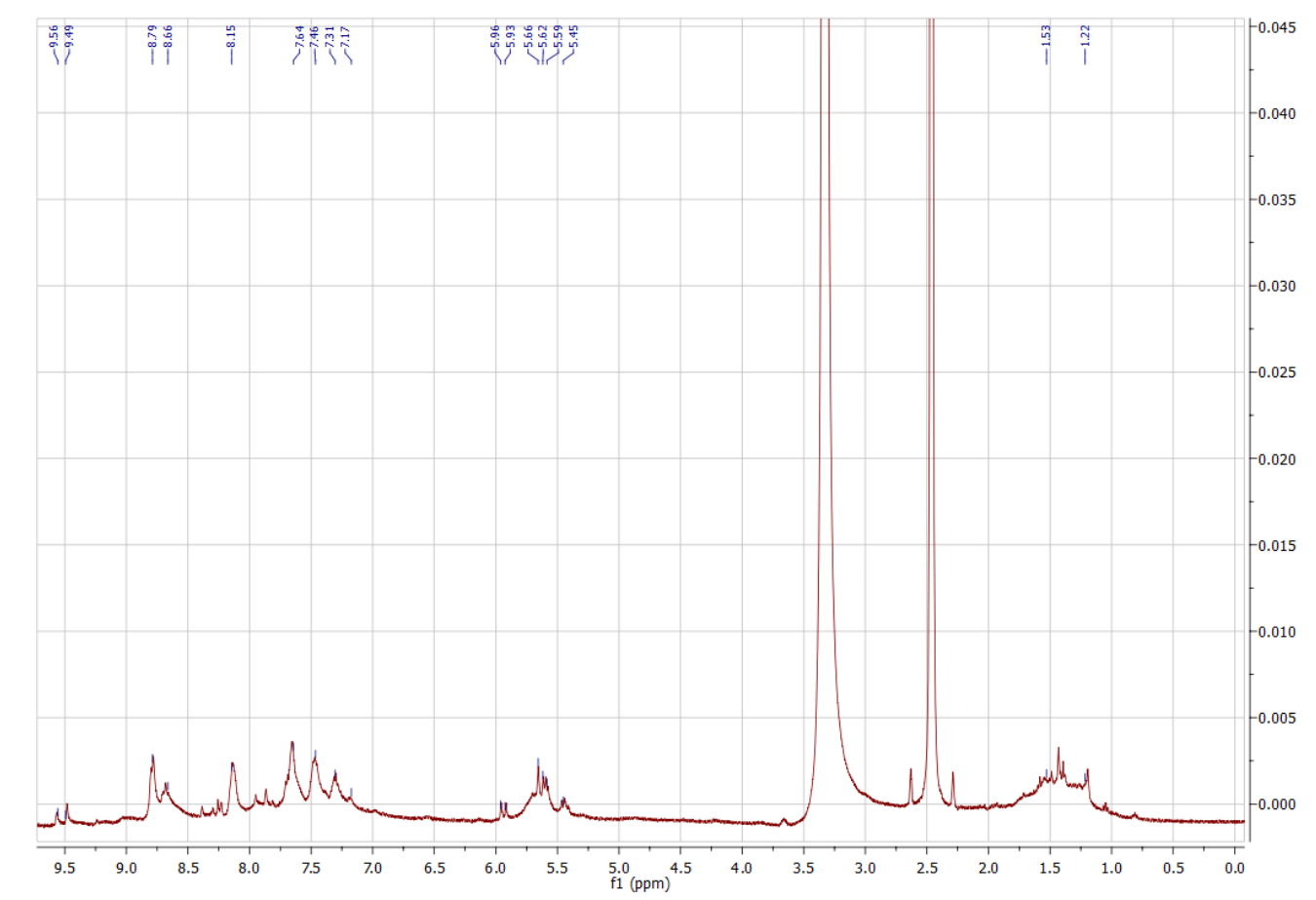


Figure S11. ^1^H NMR spectra of RR Polymer.

Table S1. ICP-MS analysis of RR Polymer

| **Sample** | **Mass ratio of Re/Ru** | **Molar ratio of Re/Ru** |
| --- | --- | --- |
| RR-polymer | 1.670704 | 0.906829018 |

Table S2. GPC analysis of RR Polymer

| **Sample** | **Mn** | **Mw** | **PDI** |
| --- | --- | --- | --- |
| RR-polymer | 468627 | 483308 | 1.03 |

**2.2 Polymer Nanoparticle Characterization Data**

Figure S12. Particle size distribution of Re NPs and Ru NPs (n=3).

Figure S13. TEM image of Re NPs and Ru NPs.


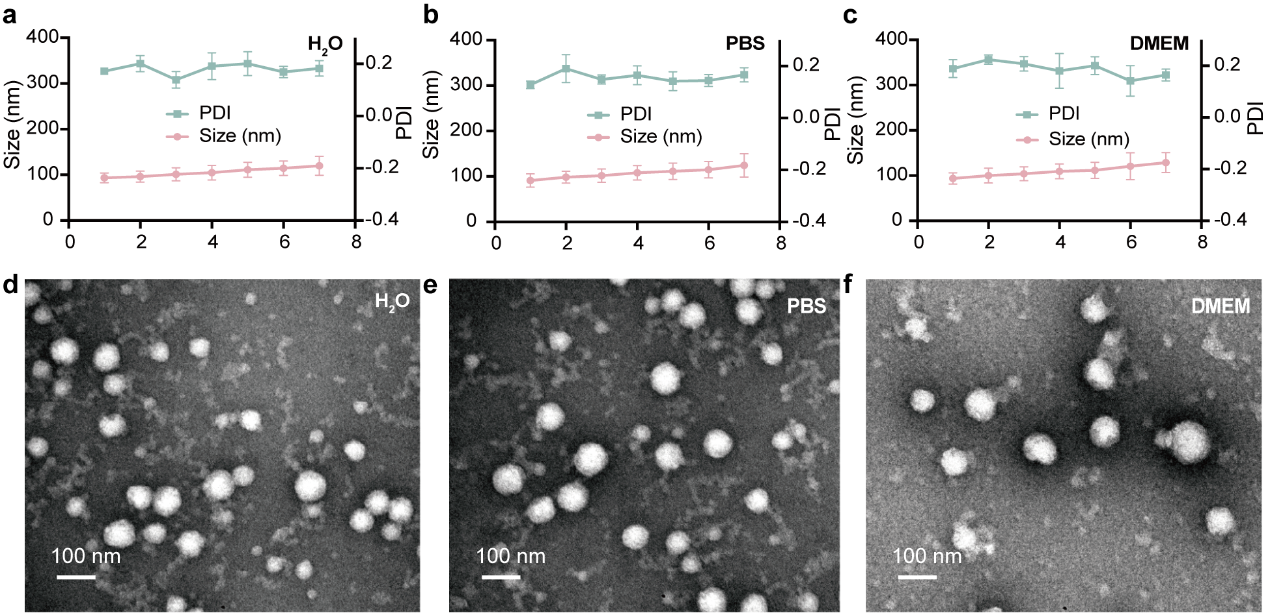


Figure S14. Hydrodynamic diameter and PDI changes of RR NPs in H_2_O (a), PBS (b), and DMEM (c) for 7 days (n = 3). TEM images of RR NPs in H_2_O (d), PBS (e), and DMEM (f) for 7 days

Figure S15. TEM image and particle size distribution (n=3) of RR NPs for one month in dark environment.

Figure S16. TEM image of Re polymer and Ru polymer blended NPs, partial phase separation between Re polymer and Ru polymer was observed.

**Figure S17.** UV spectrum of Ru NPs (30 μmol/mL), RR NPs (60 μmol/mL) and Re polymer-Ru polymer blended NPs (60 μmol/mL).

**Figure S18.** a) UV spectrum of Re NPs (30 μmol/mL) and b) Fluorescence spectrum of Re NPs.


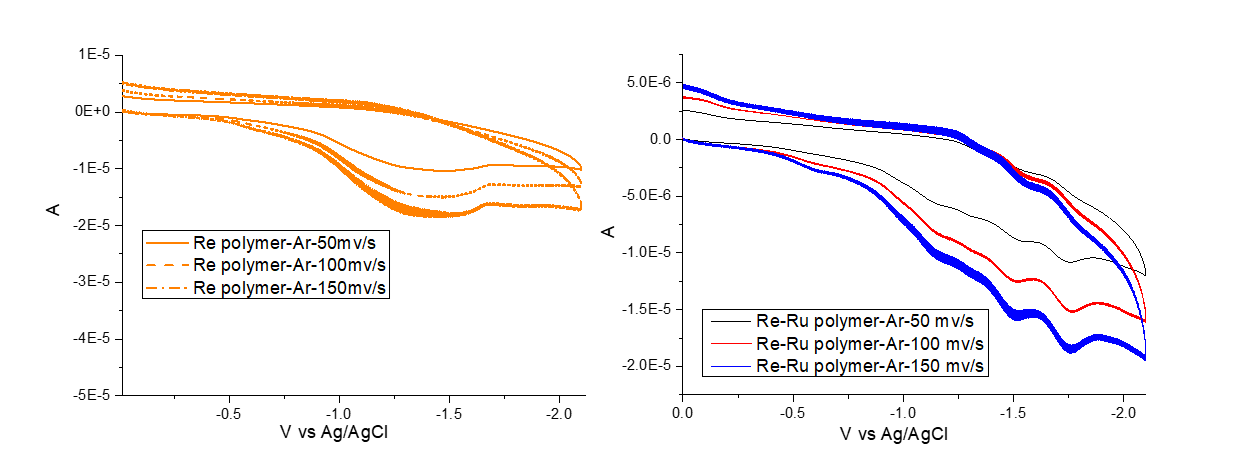


Figure S19. Redox Potential of Re polymer and RR polymer.

**2.3 *In vitro* and *in vivo* evaluation data of polymeric nanoparticles**

Figure S20. Fluorescence spectrum of Re NPs co-incubated with DCFH without (a) and with light (b).

Figure S21. UV absorption spectra of RR NPs co-incubated with myoglobin in the dark (a); Re NPs co-incubated with myoglobin in light (b) and dark (c).

Figure S22. Determination of carbon monoxide release of Re + Ru polymer blended NPs/RR NPs by gas chromatography (n=3).


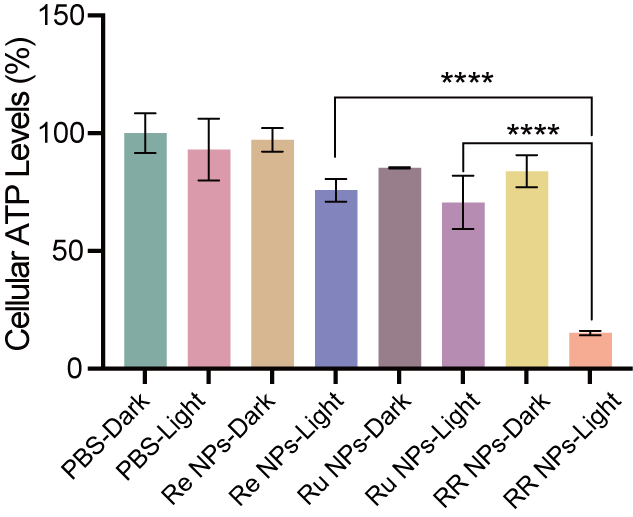


**Figure S23.** Effect of CO gas on the level of adenosine triphosphate (ATP) in HepG2 cells. ****p < 0.0001 versus Re NPs-Light group (n= 3), ****p < 0.0001 versus Ru NPs-Light group (n= 3), data were shown as mean ± SD.


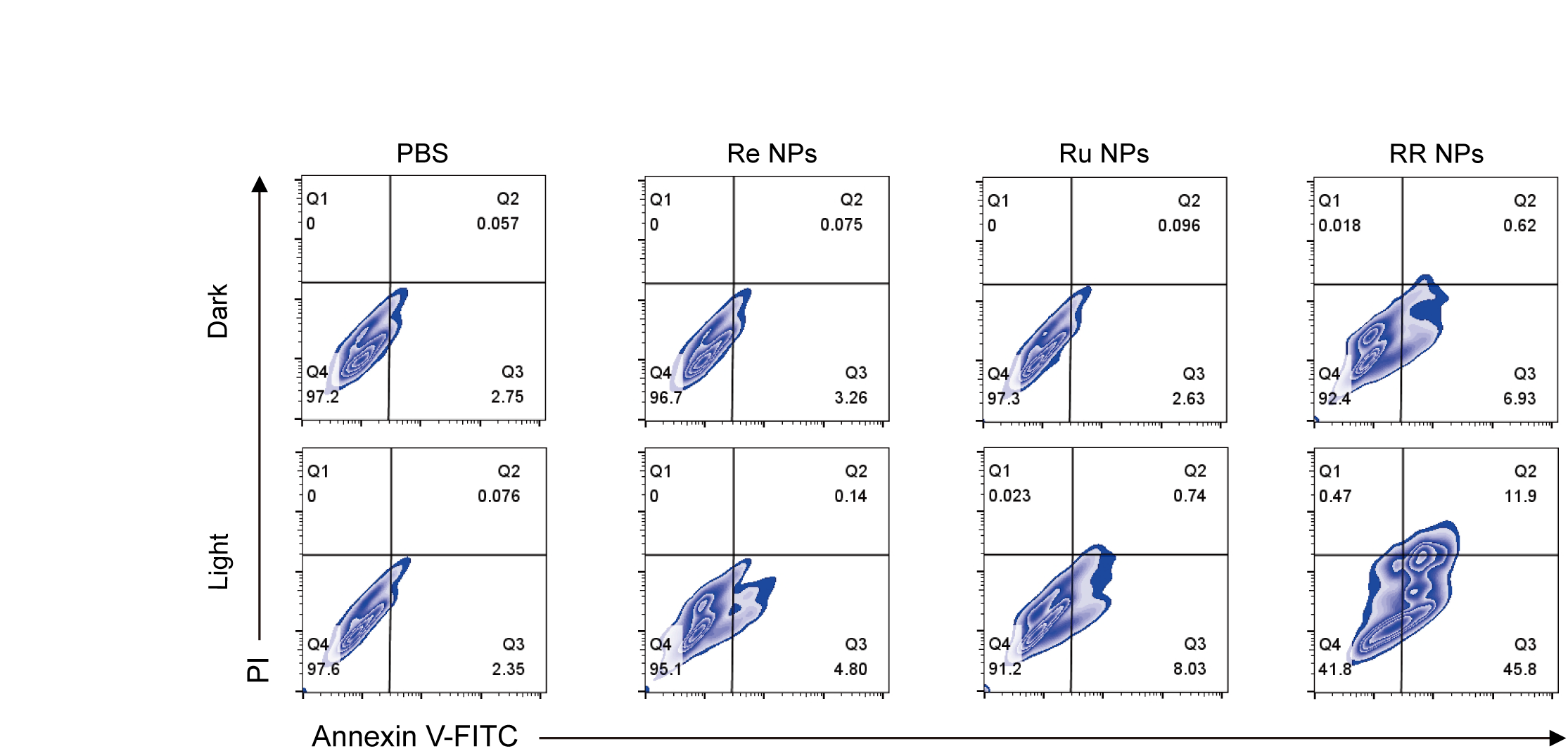


**Figure S24.** Apoptosis evaluation by flow cytometry for HepG2 cells stained with Annexin-FITC/PI under different treatment conditions.

**Figure S25.** (a) The *in vivo* biodistribution at 0-48 h post-injection of the Free-ICG group. The tumor site was circled; (b) Fluorescence imaging of excised main organs and tumors at 48 h post-injection of the Free-ICG group.

Figure S26. Averaged fluorescence intensity of excised main organs and tumors at 48 h post-injection. Data were shown as mean ± SD, ****p < 0.0001, based on one-way ANOVA.


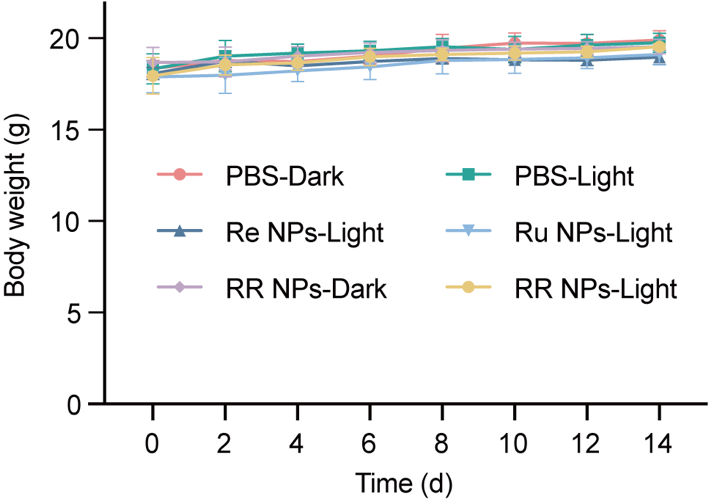


Figure S27. Body weight variations of the mice after treatment (n = 5 independent experiments)

Figure S28. Serum biochemical indexes analysis after different treatments at 15 days (n = 5). (a) TBIL, Total Bilirubin; (b) DBIL, Direct Bilirubin; (c) ALT, alanine aminotransferase; (d) ALP, Alkaline Phosphatase; (e) γ-GT, Gamma-Glutamyl Transferase; (f) UREA, Blood Urea Nitrogen; (g) CREA, creatinine; and (h) UA, uric acid.

Figure S29. Representative H&E staining images of the main organs of mice at the end of treatments.
